# Differential nitrogen assimilation by *Microcystis* and co-occurring plankton during harmful cyanobacterial blooms across North American Lakes

**DOI:** 10.3389/fmicb.2026.1794100

**Published:** 2026-04-24

**Authors:** Ann Marie E. Famularo-Pecora, Christopher J. Gobler

**Affiliations:** School of Marine and Atmospheric Sciences, Stony Brook University, Southampton, NY, United States

**Keywords:** cyanobacterial harmful algal blooms, Lake Erie, *Microcystis*, nitrogen uptake, urea

## Abstract

*Microcystis* is a toxin-producing cyanobacteria that forms colonies that host distinct microbial assemblages. This study characterized microbial diversity of, and nitrogen (N) assimilation by, isolated *Microcystis* colonies, co-occurring free-living plankton (< 20 μm), and the whole water community, in five *Microcystis* bloom-prone lakes: three larger eutrophic lakes, including Lake Erie, and two smaller, hypereutrophic systems near New York City, using high throughput sequencing of 16S rRNA and ^15^N-labeled nutrients. Bacterial and cyanobacterial community composition differed between colony and free-living fractions (*p* < 0.05), with *Microcystis* comprising the majority of cyanobacterial sequences (66–84%) within the colony fraction in four of five systems. *Microcystis* colony fractions had volumetric N uptake rates that were 2-to-22-fold higher than free-living plankton in all systems (*p* < 0.05). Urea dominated N uptake across most fractions and ecosystems, comprising 52–72% of N uptake in larger lakes and 21–52% in the smaller, hypereutrophic lakes where nitrate and ammonium were more important. N uptake rates were highly associated with lake trophic state index (TSI) as uptake rates in hypereutrophic lakes were two-to-five-fold higher than eutrophic systems. Volumetric uptake rates of urea and glutamic acid by free-living fractions were significantly and inversely correlated with TN, TP, and microcystin, suggesting blooms intensified N competition. N-specific uptake rates by colonies were significantly and inversely correlated with TN, TP, TSI, microcystin, and chlorophyll-*a*, suggesting that intensification of blooms slowed biomass-adjusted N uptake. Canonical correlation analyses (CCA) revealed *Microcystis* was associated with volumetric uptake rates of all N compounds and urea- and glutamic acid-specific uptake rates, suggesting that *Microcystis* and associated colony microbes were the dominant N assimilation pathway during blooms. This study highlights the importance of urea for *Microcystis* blooms, and demonstrates that N uptake rates of communities associated with *Microcystis* colonies differ from free-living plankton and varies as a function of lake trophic status.

## Introduction

*Microcystis* is a non-diazotrophic freshwater cyanobacterium that can cause harmful algal blooms (CHABs) and produces the hepatotoxin, microcystin. *Microcystis* CHABs have become increasingly prominent in lakes around the world (Harke et al., 2016; Ho and Michalak, 2017) and can represent a substantial public health risk (Brooks et al., 2016). *Microcystis* naturally occurs in colonies that are held together by an extracellular polysaccharide (Worm and Søndergaard, 1998). *Microcystis* colonies host dense assemblages of epiphytic and embedded bacteria (Kuentzel, 1969; Hoppe, 1981; Worm and Søndergaard, 1998; Parveen et al., 2013; Jankowiak and Gobler, 2020) that differ significantly from free-living bacterial communities (Jankowiak and Gobler, 2020). The holobiont of *Microcystis* cells and embedded bacteria, or phycosphere, is symbiotic with carbon, nutrients, and vitamins exchanged between the two components (Xie et al., 2016; Gobler and Jankowiak, 2022).

Freshwater CHABs can be promoted by the over-enrichment of nitrogen (N) and phosphorus (P) from common anthropogenic sources such as agricultural runoff and wastewater discharge (Paerl et al., 2011, 2016; Gobler et al., 2024). While it was historically presumed that P limited primary productivity and biomass in freshwater systems (Schinder et al., 2008; Paerl et al., 2016), N has recently been shown to control the biomass and/or toxicity of some CHABs (Paerl et al., 2016; Gobler et al., 2016; Hellweger et al., 2022). The relative importance of N and P can differ by ecosystem and season, with spring periods more likely to be P limited and N limitation emerging in summer (Gobler et al., 2016; Paerl et al., 2016; Chaffin et al., 2013).

N is present in freshwater systems in multiple organic and inorganic forms, most of which require enzymatic transformation before they can be incorporated into cellular biomass (Glibert, 2017). While *Microcystis* and most phytoplankton preferentially utilize ammonium which does not require enzymatic transformation (Takamura et al., 1987; Chaffin et al., 2013), organic nutrient sources including urea and amino acids (i.e., alanine) can support the growth of *Microcystis* (Belisle et al., 2016; Chaffin and Bridgeman, 2014; Newell et al., 2019). N uptake rates have been measured in non-colonial lab cultures of *Microcystis* (Chaffin et al., 2013) and using whole lake water in microcosm experiments where *Microcystis* was a dominant phytoplankton (Takamura et al., 1987; Chaffin and Bridgeman, 2014; Belisle et al., 2016). While such rates provide insight regarding the general ecophysiology of this genus, they do not represent the N assimilated by *Microcystis* colonies during blooms.

This study focused on the quantification of N assimilation by *Microcystis* colonies over space and time using a colony isolation technique. In parallel, the composition and concentration of *Microcystis* and *Microcystis* colony attached bacteria were characterized by high throughput sequencing of 16S rRNA. N uptake rates and community composition of colonies (*Microcystis* colony fraction) were compared to free-living bacteria (free-living fraction) and the total plankton community (whole water). Quantification of *in situ* nutrient concentrations, temperatures, and other environmental parameters provided contextual data for the interpretation of results. It was hypothesized that *Microcystis* colonies (>20 μm) and free-living bacteria (< 20 μm) would have (1) significantly different community composition and (2) differing uptake rates and relative preferences for different N sources with a preference for ammonium within the *Microcystis* colony fraction.

## Materials and methods

### Study sites and collection

**Figure 1:**
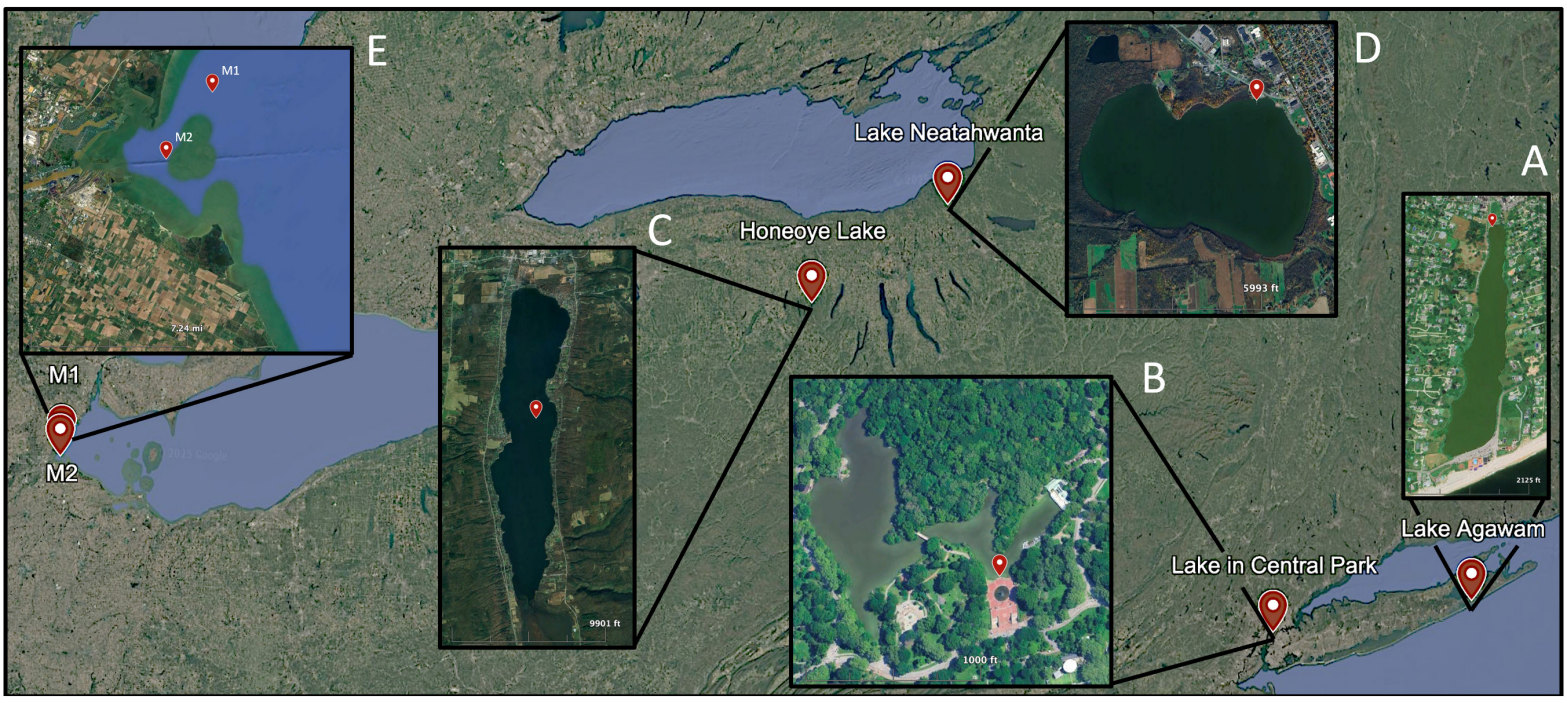
Sampling locations: **(A)** Lake Agawam time series study site. **(B)** The Lake in Central Park time series study site. **(C)** Honeoye Lake study site. **(D)** Lake Neatahwanta study site. **(E)** Lake Erie study sites located across the Maumee Bay region (M1–M2) in the western basin. Site M1 was sampled in 2021 and site M2 was sampled in 2022.

Water was collected between June and October during 2020, 2021, and 2022 from five North American lakes including Lake Agawam (LA; 40.88148,−72.39256; Figure 1A), the Lake in Central Park (LCP; 40.77458,−73.97073; Figure 1B), Honeoye Lake (HE; 42.75582,−77.50968; Figure 1C), Lake Neatahwanta (NT; 43.31385,−76.42956; Figure 1D), and western Lake Erie (LE; M1: 41.72695,−83.40376; M2: 41.78125,−83.39589; Figure 1E), all locations prone to dense blooms of *Microcystis* (Jankowiak and Gobler, 2020; Jankowiak et al., 2019; Hellweger et al., 2022). The Lake in Central Park and Lake Agawam were sampled weekly to monthly during this period while the other water bodies were sampled one or more days during *Microcystis* bloom events. On site, surface conditions including temperature, dissolved oxygen, and pH were measured with a YSI 556 ProQuatro multiparameter sonde and a 20 L sample of surface water was collected and transported in a polycarbonate carboy to laboratories for analyses. *In vitro* chlorophyll-*a* concentrations of planktonic brown algae, chlorophytes, and cyanobacteria were measured using a bbe Moldaenke Fluoroprobe (Beutler et al., 2002). Concentrations were adjusted utilizing the difference between *in vitro* concentrations and extracted chlorophyll-*a* (*analytical details below*). Dominant cyanobacterial genera were identified using an inverted Nikon Eclipse TS100 microscope via a gridded 1 mm^2^ Sedgewick Rafter counting chamber. Total (whole water) and dissolved (filtered through an EMD Millipore APFB 0.7 μm pore size glass fiber filter, combusted at 450°C for 2 h) nutrient samples were collected in duplicate and stored at −20°C until further processing. Nutrient samples were analyzed for nitrate, ammonia, urea, total nitrogen, phosphate, and total phosphorus on a Lachat Instruments flow injection autosampler (ASX-520 series) using standard wet chemical methods (Valderrama, 1981; Jones, 1984; Parsons, 2013); Analyses proceeded once completed recovery of standard reference material for ammonia, nitrate, phosphate, total N and total P was achieved (SPEX CertiPrep™ standards). Nutrient samples were also analyzed for glutamic acid using an Agilent 6495B triple quadrupole liquid chromatography tandem mass spectrometer (LC-MS/MS) equipped with electron spray ionization (ESI) using standard hydrophilic interaction chromatography for plant matrices (Huang et al., 2018). Concentrations of nitrate, ammonia, urea, and glutamic acid were utilized in N uptake calculations (*analytical details below)*. Duplicate total (whole water) microcystin samples were collected and analyzed by an ABRAXIS^®^ Microcystin/Nodularians test kit according to the manufactures' (Gold Standard Diagnostics) recommended procedures.

### *Microcystis* colony isolation

Lake water (whole water) was separated into two planktonic fractions: *Microcystis* colonies and free-living bacteria, by utilizing an approach from Jankowiak and Gobler (2020). Briefly, 1–4 L (dependent on cyanobacterial density) of well-mixed lake water was passed through a 20 μm mesh sieve, resulting in filtrate of free-living bacteria (< 20 μm), and concentrate which includes *Microcystis* colonies, embedded bacteria, and other particulates; utilizing the operational definition of a *Microcystis* colony by Worm and Søndergaard (1998). The concentrate was resuspended in 250 mL of 0.2 μm filtered lake water. *Microcystis* colonies, which are buoyant, and other attached particulates were then allowed to separate from non-buoyant particulate matter after a short period of time. Once separated, *Microcystis* colonies were removed from the top layer with a serological pipette and resuspended twice more into 250 ml of 0.2 filtered lake water within ~10 min to further isolate colonies from non-colony particles and plankton. Isolated colonies were volumetrically diluted to the original volume initially passed over the sieve with 0.2 μm filtered lake water back to the densities present prior to concentrating this fraction on the 20 μm mesh. An aliquot of this fraction was examined via microscopy to confirm there was no overt contamination of large particles or non-Microcystis phytoplankton. Each fraction, as well as whole water and the remaining non-buoyant solution, were analyzed on a bbe Moldaenke Fluoroprobe for algae class quantification, and preserved in Lugols iodine solution (5% v/v) for microscopic analysis on a Keyence BZX-800 that revealed that the abundance and size distribution of the *Microcystis* colonies in the isolated *Microcystis* colony fraction were highly similar to and not different from those in the whole water samples (Gobler et al., 2026) indicating the colony matrix integrity was not altered during this process. The term “*Microcystis* colony fraction,” therefore, is an isolated sample of *Microcystis* cells and embedded bacteria, as seen in previous studies (Parveen et al., 2013; Jankowiak and Gobler, 2020). Each fraction was filtered in triplicate onto a pre-combusted (450 °C for 2 h) Millipore APFB 0.7 μm pore size glass fiber filter for measurement of particulate organic N concentration (PON; >0.7 μm) and δ^15^N content (*analytical details below*). For 16S rRNA analyses, triplicate samples (25–50ml) of whole water, the free-living fraction (< 20 μm), and isolated *Microcystis* colony fraction were filtered onto 0.22 μm polycarbonate filters. All 16S rRNA samples were immediately frozen in liquid N and stored at −80°C until DNA extraction.

### Nitrogen (^15^N) uptake rates

Tracer experiments were conducted with ^15^N-labeled compounds to determine net uptake rates by whole water, the free-living fraction, and *Microcystis* colony fraction. Uptake rates of nitrate, ammonium, urea, and glutamic acid were measured using additions of highly enriched (≥98%) ^15^N-nitrate, ^15^N-ammonium, ^15^N-urea, and ^15^N-glutamic acid (Mulholland et al., 2002; Harke and Gobler, 2015) at a target concentration of ~10% of previously measured *in situ* concentrations within the ecosystems (Chaffin et al., 2013, 2014; Davis et al., 2010; Jankowiak and Gobler, 2020; Jankowiak et al., 2019). The target percent (10%) of ambient N concentrations minimizes over enrichment, maintains isotopic integrity, and ensures detectability of the tracer concentration (Dugdale and Goering, 1967; Glibert et al., 1993; Knap et al., 1996; Elskens et al., 2005). Actual additions averaged 8.6 ± 1.2% of *in situ* nutrient concentrations being 7.1 ± 1.6% for nitrate, 2.5 ± 0.5% for ammonium, 0.3 ± 0.03% for urea, and 24.3 ± 2.5% for glutamic acid. Whole water and the *Microcystis* colony fraction were distributed into triplicate 50 ml polycarbonate culture flasks and incubated for 60 min at *in situ* lake water temperature and ambient light levels. This shorter incubation time was utilized to minimize ^15^N recycling and complications due to isotopic fractionation (Elskens et al., 2005). After 60 min, half of the whole water samples were further isolated using a 20 μm sieve and filtrate was collected on combusted glass fiber filters (GFF) to represent the free-living fraction samples; The other half of the whole water samples and the *Microcystis* colony fraction were filtered directly onto GFF filters, and frozen. GFF filters were dried (24 h @ 60 °C), pelleted in tin discs, and analyzed by the U.C. Davis Stable Isotope Facility (Davis, CA) via an Elementar vario Micro Cube elemental analyzer (Elementar Analysensysteme GmbH, Hanau, Germany) interfaced to a PDZ Europa 20–20 isotope ratio mass spectrometer (Sercon Ltd., Cheshire, UK). Samples were corrected based on batch-specific calibrated reference materials and final δ^15^N values are expressed relative to international air standards for N.

Calculated uptake rates utilized within this study include (1) volumetric N uptake, (2) N specific uptake, and (3) N relative preference. Volumetric N uptake rates (V, μmol L^−1^ h^−1^) by the *Microcystis* colony fraction, free-living fraction (< 20 μm), and the whole water community were quantified according to Glibert et al. (1982):


V≈(N15s−NAN15Enr−NA)PONT

NA, ^15^*N_Enr,_* and ^15^*N_s_* are the atom % abundances of ^15^N of the cells prior to incubation, of the substrate (e.g., nitrate, etc.) after isotopic enrichment, and of the cells after the incubation, respectively, *T* is the incubation time, and PON is the particulate N per volume in samples. Rates were not corrected for the effects of isotope dilution (Glibert et al., 1982) and therefore are considered net uptake; however, uptake, release, and subsequent re-uptake of compounds during incubations were likely limited given the brief incubation period (60 min). N specific uptake (day^−1^), also known as biomass weighted uptake, by *Microcystis* colonies, free-living, and whole water communities were calculated per sample date and site by dividing the volumetric uptake rates (*V*) by the PON concentrations for each fraction within each sample. N relative preference (%) by the *Microcystis* colony fraction, free-living fraction, and whole water communities were calculated per sample date and site by dividing individual volumetric uptake rates (*V*) by the total volumetric uptake of all N forms, then adjusted to a percentage. Differences in rates and relative preference by lake and for the entire study were assessed via two-way ANOVA's where plankton group (whole, colony, free-living) and N form were the treatment factors with *post-hoc* Tukey tests using SigmaPlot (Version 15). If rates were not normally distributed, determined through a Shapiro-Wilk test, a two-way ANOVA on ranks (Kruskal-Wallis) was performed in R (version 4.4.1).

### DNA isolation, sequencing, and analysis

Samples for 16S rRNA sequencing were extracted with a DNAasy^®^ Power Water Kit (QIAGEN), modified with an additional lysing step for cyanobacteria, and the quality of DNA was evaluated on a NanoDrop Microvolume Spectrophotometer (ThermoFisher) and the quantity of DNA in extracts was evaluated on a Qubit Fluorometer (ThermoFisher), allowing the concentration of DNA for sequencing to be normalized across all samples. Samples from 2021 and 2022 were sent to the Molecular Research Laboratories in Shallowater, TX, for 16S V4 universal sequencing at 20,000 reads per sample; 2020 samples were lost. The 16S rRNA gene was amplified using the 515F/806R primer set and used to distinguish between cyanobacterial genera.

After identifying barcodes were placed on the forward primers for each sample, PCR amplification was performed using the HotStarTaq Plus Master Mix Kit (QIAGEN) with the cycling conditions as follows: 1 cycle at 95 °C for 5 min, 30 cycles at 95 °C for 30 s, 1 cycle at 53 °C for 40 s, 1 cycle at 72 °C for 1 min, and a final elongation step at 72 °C for 10 min. 2% agarose gel was then utilized to visualize the success of the amplification and the relative intensity of the PCR products. Following confirmation of amplification, samples were multi-plexed using unique dual indices and then pooled together in equal proportions based on their molecular weights and DNA concentrations. Samples were then purified using calibrated Ampure XP beads (Beckman Coulter Life Sciences) and used to prepare a DNA library for paired end reads (20 K; 2 × 300) with an Illumina MiSeq platform. All sequences have been deposited in Genbank under accession number PRJNA1356834.

Sequences were processed using the Quantitative Insights Into Microbial Ecology QIIME 2 (version 2021.4.0) microbiome analysis software package following the “Moving pictures” pipeline (Bolyen et al., 2019). Briefly, paired-end reads were trimmed of their primers and barcodes using the Cutadapt plugin (Martin, 2011), and then merged by Dada2 to produce a table of exact (100%) amplicon sequence variants (ASV; Callahan et al., 2016). This collectively generated a total of 17,173,808 sequences after joining and quality filtering with an average length of 253 bp between 2021 and 2022. The sequences clustered into 16,766,251 total ASVs, containing 30,713 unique ASV's with 100% sequence identity considered for analysis, which contained 28,507 unique bacterial ASVs, and 1,342 unique cyanobacterial ASV; Among the total ASV's, 407,557 mitochondrial/chloroplast ASVs were not considered for further analysis. Taxonomic identification of the 16S dataset was achieved using the 99% 16S only rep set FASTA and majority consensus seven-level taxonomy files of the SILVA rRNA (16S SSU) release v138 database (Quast et al., 2012), which characterized the composition of the three plankton groups. Sequences within each plankton group were evaluated for variance between groups within QIIME 2 (version 2021.4.0) by principal coordinate analysis (PCoA) and for evenness and richness by Shannon richness and Pielou's evenness.

### Analysis of environmental and biological drivers

Physio-chemical parameters (lake area, lake depth, fluoroprobe-derived algal biomasses, lake surface temperature, N concentrations, and microcystin concentrations) were compared with N uptake rates using Mantel tests and Spearman correlation matrices in R (version 4.4.1). If parameters were not normally distributed, visualized through histogram analysis, parameters were normalized by natural log transformation prior to correlation analyses. Additionally, multivariate statistical approaches were employed to analyze differences among the 16S community structure (composition/abundance) in relation to N uptake rates in R (version 4.4.1). Specifically, similarity percentage analysis (SIMPER) was conducted to identify taxa driving the community dissimilarity between plankton groups and canonical correspondence analyses (CCA) was performed after normalization to identify potential drivers of, and associations between, community compositions and N uptake. CCA analyses were performed in R (version 4.4.1) utilizing the picante software package (Kembel et al., 2010) and Biodiversity analysis in R pipeline (Kembel, 2011).

Finally, the Trophic State Index (TSI) was calculated for each system using Secchi disc depths, chlorophyll-*a*, total phosphorus, and orthophosphate following equations from Toledo et al. (1983) as described by Klippel et al. (2020). Trophic State Index (TSI) is a numerical system that uses environmental parameters to rank ecosystems from hypereutrophic to ultraoligotrophic on a scale from 0 to 100 (Carlson, 1977; Toledo et al., 1983; Toledo and Companhia de Tecnologia de Saneamento Ambiental (CETESB), 1990; Paulic et al., 1996; Klippel et al., 2020). TSI was utilized to directly compare ecosystem productivity between all waterbodies within this study. Given the differences between site morphology and hydrology, TSI was viewed as a comparative index of trophic condition and correlations were interpreted as associative relationships.

## Results

### Physiochemical conditions and cyanobacteria bloom characteristics

**Figure 2:**
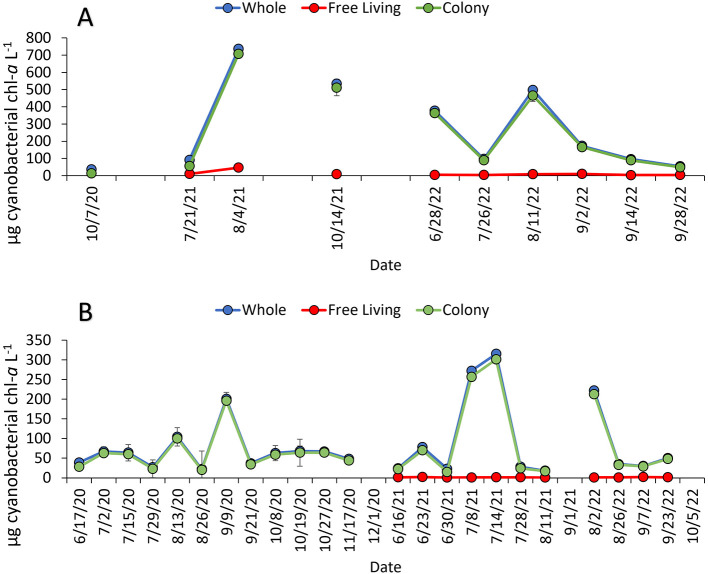
The average cyanobacterial concentration of whole water and fractions from **(A)** the Lake in Central Park time series study site and **(B)** the Lake Agawam time series study site from summers of 2020–2022. All values were corrected by the average extracted particulate value. Points are means and error bars are ±1 S.D. Error bars are often smaller than data points.

**Figure 3:**
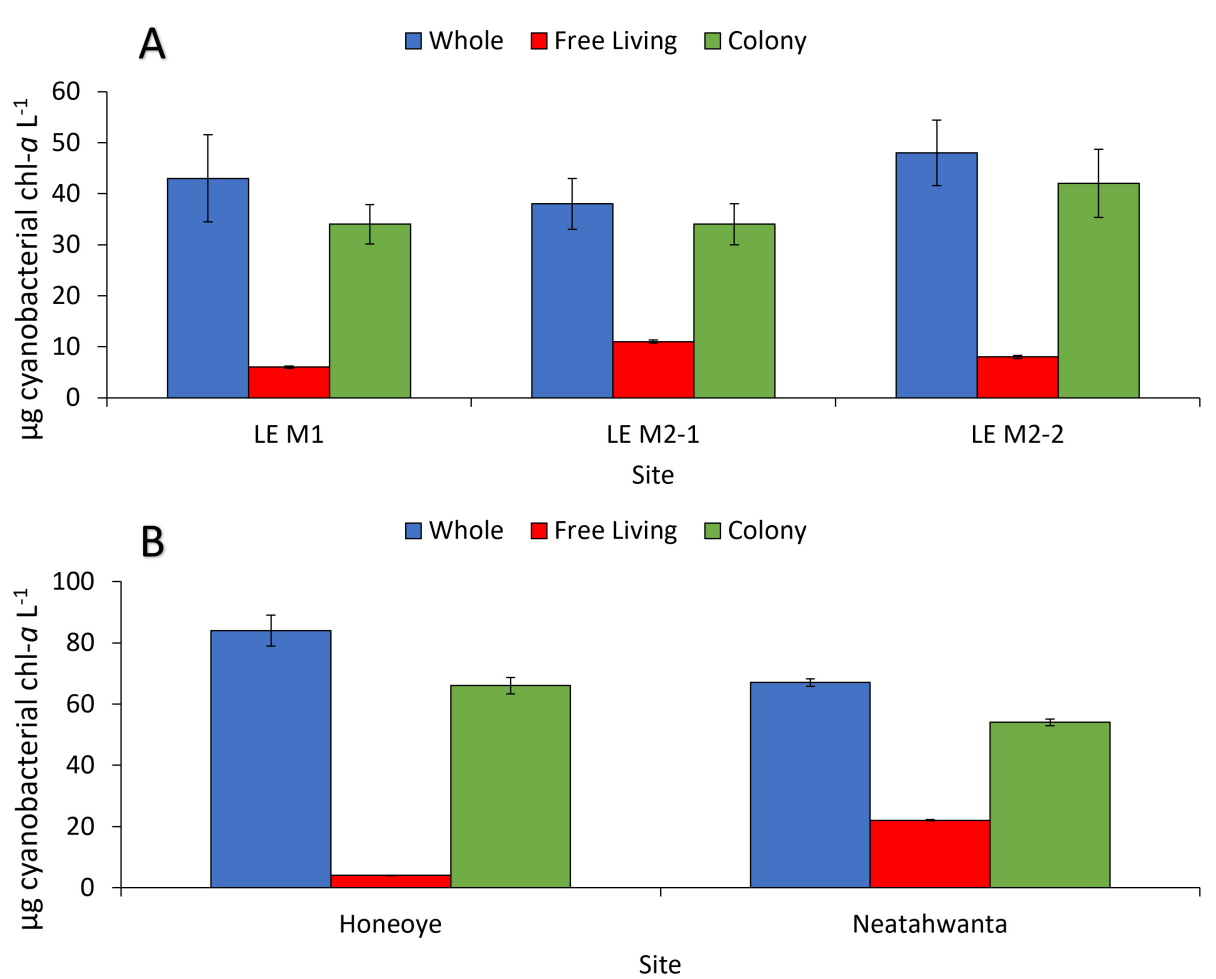
The average cyanobacterial concentration of whole water and fractions from **(A)** the Lake Erie transect study sites from summers of 2021 (M1) and 2022 (M2), and **(B)** Honeoye Lake (9/2021) and Lake Neatahwanta (9/2021). Site M2 was sampled on two consecutive dates in 2022: 8/18/2022 (M2-1) and 8/19/2022 (M2-2). All values were corrected by the average extracted particulate value. Points are means and error bars are ±1 S.D.

**Figure 4:**
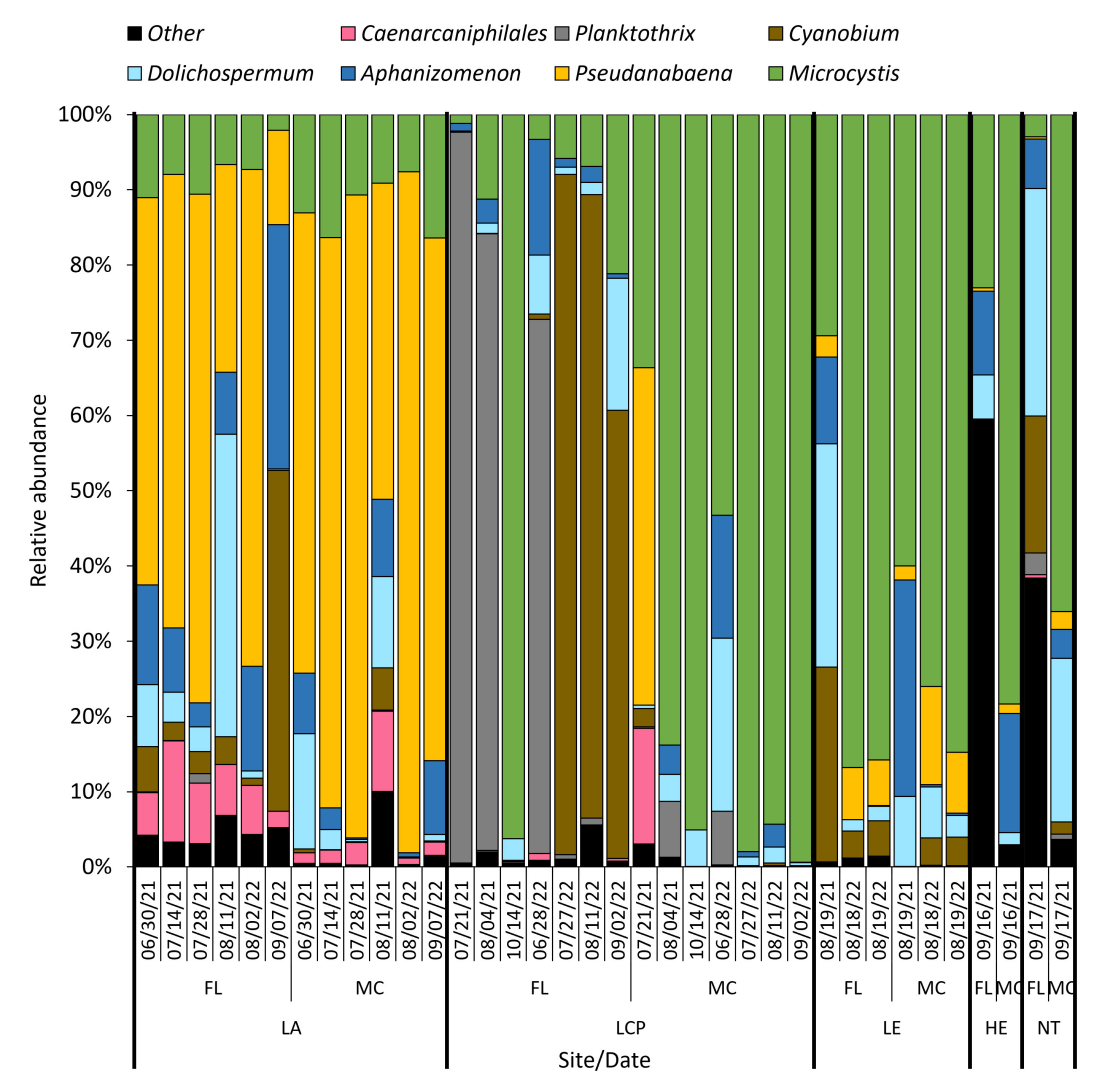
Time series of the cyanobacterial relative abundance determined via 16S sequencing in the free-living bacteria (FL) and *Microcystis* colony (MC) fractions, grouped by lake (LA, Lake Agawam; LCP, The Lake in Central Park; LE, Lake Erie; HE, Honeoye Lake; and NT, *Lake Neatahwanta*) All low abundant phyla have been grouped into the “other” category.

All study sites hosted dense cyanobacterial blooms (50–7,300 μg cyanobacteria-chl-*a* L^−1^) during summer and fall of 2020–2022 (Figures 2, 3). Across sites, whole water and the *Microcystis* colony fraction contained the highest concentration of cyanobacteria (60–730 μg cyanobacteria-chl-*a* L^−1^ and 50–710 μg cyanobacteria-chl-*a* L^−1^, respectively), and PON (3–11 mg N L^−1^ and 2–10 mg N L^−1^, respectively), while the free-living fraction had the lowest biomass and PON (1–50 μg cyanobacteria-chl-*a* L^−1^ and 1–3 mg N L^−1^, respectively; Supplementary Figures 1, 2). Lake Neatahwanta was the exception with similar cyanobacterial biomass and PON concentrations measured within both the free-living and *Microcystis* colony fractions (Figure 3B; Supplementary Figure 2B). *Microcystis* dominated cyanobacterial communities for the whole water, free-living fraction, and *Microcystis* colony fraction, comprising on average 72 ± 8%, 31 ± 10%, and 73 ± 7% of the relative frequency for cyanobacterial ASV's, respectively. This excludes Lake Agawam which was predominately *Pseudanabaena*, comprising 50 ± 10%, 48 ± 10%, and 76 ± 5% of those ASVs, respectively (Figure 4; Supplementary Figure 3; *further details below*). The dominant form of dissolved inorganic and organic nitrogen varied among lakes with ammonium, nitrate, and urea representing the largest pool depending on site. Specifically, ammonium was dominant in the Lake in Central Park (0.09 ± 0.03 mg N L^−1^), nitrate in Lake Erie (0.3 ± 0.2 mg N L^−1^) and Lake Agawam (0.06 ± 0.02 mg N L^−1^), urea in Lake Neatahwanta (0.04 mg N L^−1^) and nitrate and urea having similar concentrations in Honeoye Lake (0.02 mg N L^−1^ for both; Table 1). For sites with seasonal measurements (Lake Agawam, Lake in Central Park), concentrations of nitrate and ammonium were often higher in spring and fall (Table 1). Total nitrogen (TN) and total phosphorous (TP) were highest in the Lake in Central Park (11.0 ± 3.1 mg N L^−1^ and 2.2 ± 0.92 mg P L^−1^) and Lake Agawam (4.9 ± 1.1 mg N L^−1^ and 590 ± 520 μg P L^−1^) with lower concentrations in Lake Erie (2.3 ± 0.2 mg N L^−1^ and 120 ± 10 μg P L^−1^), Honeoye Lake (1.4 mg N L^−1^ and 238 μg P L^−1^), and Lake Neatahwanata (1.7 mg N L^−1^ and 89.8 μg P L^−1^). The TSI classified the Lake in Central Park (92.8) and Lake Agawam (78.8) as hypereutrophic and Lake Erie (69.2), Honeoye Lake (72.9), and Lake Neatahwanta (67.5) as eutrophic.

**Table 1:** *In situ* physiochemical conditions and nutrient concentrations during the Lake Agawam (LA) time series, the Lake in Central Park time series (LCP), the Lake Erie (LE) transects (sites M1–M2), and individual sampling sites for Honeoye Lake (HE) and Lake Neatahwanta (NT).

Site	Date	Surface temperature (°C)	Microcystin (μg L^−1^)	Nitrate (μM)	Ammonia (μM)	Urea (μM)	Glutamic acid (μM)	Orthophosphate (μM)	TN (μM)	TP (μM)
LA	6/17/20	22.1	11	17.63	9.28	1.41	0.01^*^	0.67	–	–
LA	7/2/20	27.1	27	0.21	0.40	1.06	0.01^*^	6.05	257.82	8.02
LA	7/15/20	26.0	35	0.56	0.99	0.85	0.01^*^	3.33	239.83	11.44
LA	7/29/20	29.9	23	0.67	0.59	1.11	0.01^*^	1.67	–	–
LA	8/13/20	28.9	103	0.12	1.16	1.25	0.01^*^	–	401.12	–
LA	8/26/20	25.1	12	0.81	1.65	2.43	0.01^*^	2.72	153.73	6.85
LA	9/9/20	25.7	94	0.18	0.49	1.26	0.01^*^	–	–	–
LA	9/21/20	16.2	21	0.55	0.63	0.64	0.01^*^	–	–	–
LA	10/8/20	16.5	59	0.49	1.13	–	0.01^*^	–	–	–
LA	10/19/20	14.9	33	2.32	0.68	1.20	0.01^*^	0.73	–	–
LA	10/27/20	15.0	49	0.38	0.70	1.60	0.57	0.81	–	–
LA	11/17/20	13.3	22	10.09	11.73	1.40	0.67	0.83	–	–
LA	6/16/21	23.3	36	32.03	6.42	1.17	–	0.90	191.64	4.01
LA	6/23/21	22.7	60	0.99	1.01	0.65	–	0.26	275.17	7.77
LA	6/30/21	26.5	30	0.30	1.15	2.04	–	0.27	112.55	5.44
LA	7/8/21	26.3	274	0.00	0.71	0.96	–	0.17	1,130.10	41.05
LA	7/14/21	23.7	244	1.23	1.00	1.24	0.034^*^	0.19	765.76	32.87
LA	7/28/21	27.0	25	1.30	0.89	1.07	0.034^*^	0.21	173.01	6.38
LA	8/11/21	25.3	26	–	–	–	–	–	–	–
LA	8/2/22	24.9	218	1.63	9.60	2.13	0.071	3.51	675.16	50.50
LA	8/26/22	26.9	28	1.56	1.96	0.75	0.072	2.42	186.90	8.02
LA	9/7/22	23.0	22	3.19	3.18	1.28	0.074	0.68	154.34	5.60
LA	9/23/22	19.8	31	3.28	1.80	0.66	0.069	0.79	222.02	7.46
LA	10/5/22	14.3	28	7.71	1.80	0.00	0.067	0.36	121.55	4.29
LCP	10/7/20	17.7	8	–	–	–	–	–	–	–
LCP	7/21/21	27.2	8	1.02	1.01	1.46	0.034^*^	0.71	146.67	10.11
LCP	8/4/21	27.2	150	0.86	1.53	3.31	0.078	0.80	1,305.50	62.32
LCP	10/14/21	18.9	503	3.15	1.02	0.74	0.089	0.77	1,390.31	–
LCP	6/28/22	24.3	127	2.60	10.99	2.64	0.068	2.73	810.10	44.24
LCP	7/27/22	27.0	35	3.76	3.82	0.42	0.059	0.69	272.77	19.12
LCP	8/11/22	29.7	130	5.28	22.45	1.85	0.113	0.40	2,051.48	–
LCP	9/2/22	25.3	45	2.36	12.99	1.66	0.109	2.33	667.45	34.71
LCP	9/14/22	23.4	29	1.53	3.27	2.94	0.072	1.47	267.42	13.38
LCP	9/28/22	18.7	7	3.65	1.62	0.95	0.079	1.33	162.48	9.65
LE M1	8/19/21	25.9	–	44.58	0.60	1.51	0.315	0.58	196.14	3.55
LE M2	8/18/22	23.0	11	19.38	1.35	2.80	0.226	0.28	157.34	4.37
LE M2	8/19/22	23.6	8	1.06	1.77	2.08	0.226	0.46	141.06	3.41
HE	9/16/21	22.0	2	0.34	1.40	0.69	0.085	0.27	102.55	7.45
NT	9/17/21	21.6	10	2.17	1.01	1.30	0.034^*^	0.31	121.44	2.81

^*^BDL, value notated is half of the detection limit.

### Microbial sequencing

Samples from 18 sites/dates (2021 and 2022) were sequenced, generating 54 samples from the whole water and two fractions (plankton groups). Among all samples, >28,000 unique heterotrophic bacterial ASVs were identified via 16S rRNA sequencing. *Microcystis* colony and associated bacterial communities were significantly different than whole water communities (2022: *p* < 0.05; PERMANOVA) and free-living communities (2021: *p* = 0.003, 2022: *p* = 0.001; PERMANOVA; Supplementary Figures 4A, 5A, B; Supplementary Table 1). The *Microcystis* colony fraction was significantly less diverse than the free-living fraction and whole water communities in both 2021 and 2022 (*p* < 0.05 and *p* < 0.001, respectively; PERMANOVA; Supplementary Figures 4A, 5A, B). Whole water and its subset fractions explained the largest amount of variability between both years at 26% and 31% for 2021 and 2022, respectively (Supplementary Figures 4A, 5A, B; Supplementary Table 1). Additionally, lake systems explained 16% of variability regarding heterotrophic bacterial communities (*p* = 0.001 for both years; PERMANOVA; Supplementary Figures 4B, 5C, D; Supplementary Table 1). *Proteobacteria* and *Bacteroidota* were the dominant heterotrophic bacterial phyla in all plankton groups (whole, free-living, and *Microcystis* colony; Supplementary Figures 6, 7). Cyanobacterial communities (1,342 unique ASV's) were significantly different between plankton groups in both years (*p* < 0.05; Tukey) with free-living and *Microcystis* colony fractions being the most dissimilar (*p* = 0.002). The *Microcystis* colony fraction was significantly less rich and less even compared to the free-living fraction for both the Lake in Central Park and Lake Agawam (*p* < 0.001 for both). Specifically, *Microcystis* was significantly more abundant in the *Microcystis* colony fraction compared to the free-living fraction (*p* < 0.05; ANOVA; Figure 4) and all other abundant genera were significantly more abundant in the free-living fraction compared to the *Microcystis* colony fraction (*p* = 0.001; ANOVA; Figure 4). Overall, these subset fractions contained the most dissimilarity driven by *Microcystis* (34.32%), *Pseudanabaena* (21.55%), and *Cyanobium* (15.79%; SIMPER).

### Nitrogen uptake

Across the entire study, N uptake was consistently different for both N source and plankton group (whole, colony, free-living). All uptake rates, including volumetric, N-specific, and preference, differed significantly among all N sources (*p* < 0.001 for all), while, by plankton group, volumetric N uptake rates (*p* < 0.001) and N-specific uptake rates (*p* < 0.05) were significantly different, but relative preference was not (*p* = 0.897).

**Figure 5:**
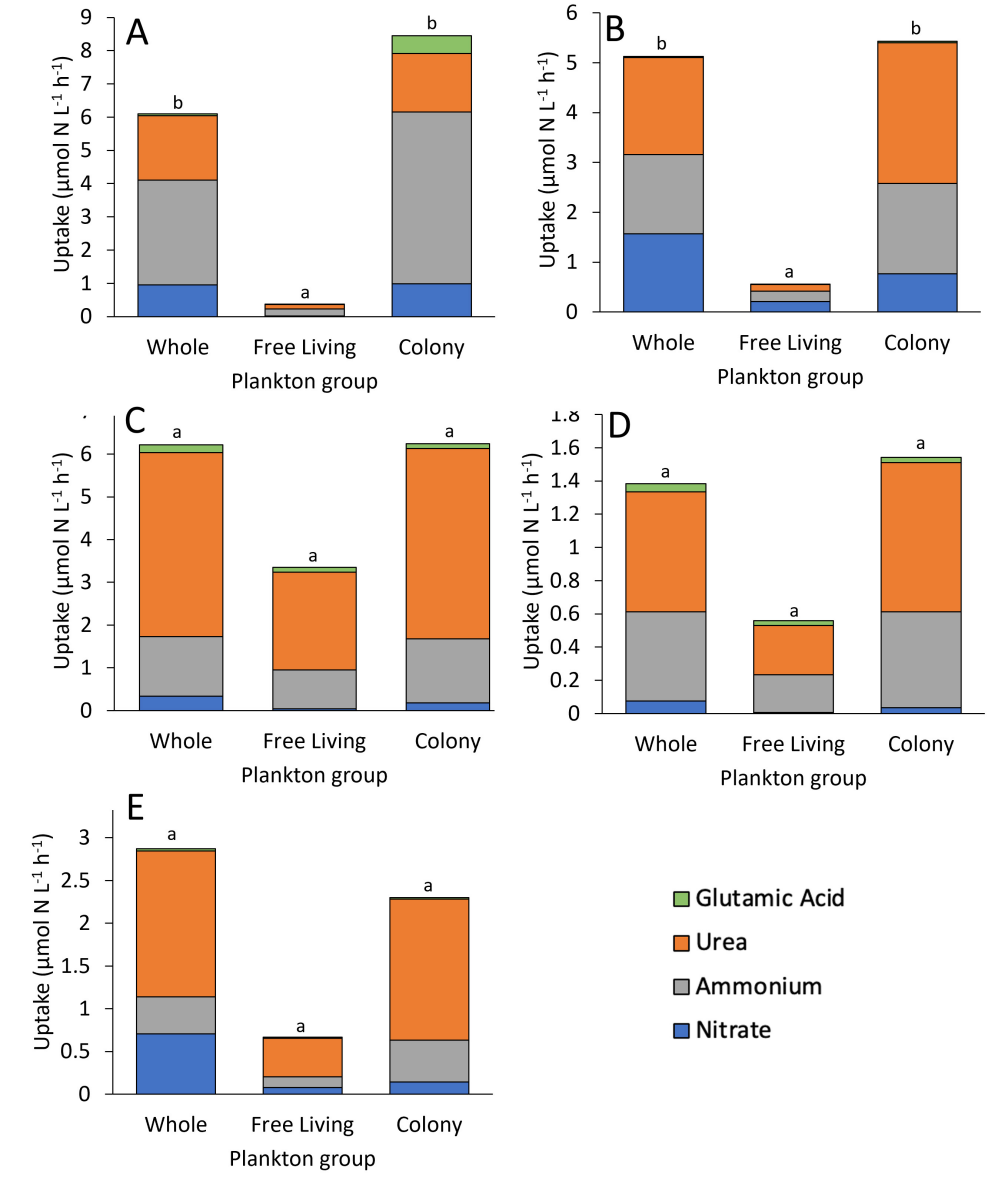
Mean volumetric nitrogen uptake of whole water and fractions for **(A)** the Lake in Central Park, **(B)** Lake Agawam, **(C)** Lake Erie, **(D)** Honeoye Lake, and **(E)** Lake Neatahwanta, including nitrate, ammonium, urea, and glutamic acid. All systems had higher uptake in *Microcystis* colony fraction than free-living fraction, with highest uptake of urea for all sites (*p* < 0.05), excluding **(A)** the Lake in Central Park and **(B)** Lake Agawam where ammonium had higher uptake (*p* < 0.05). Letter denotations indicate significance between water groups.

**Figure 6:**
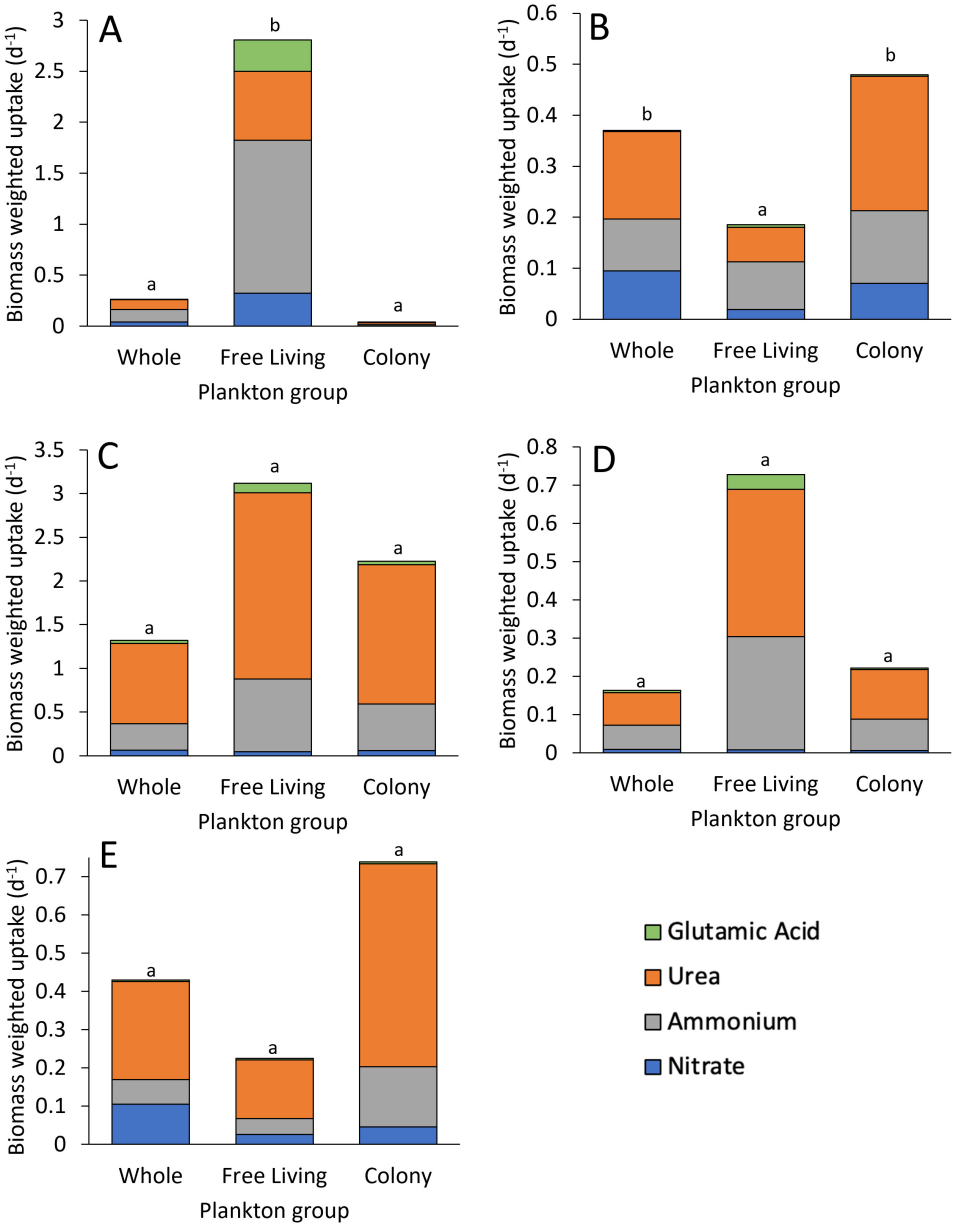
Mean nitrogen-specific uptake of whole water and fractions for **(A)** the Lake in Central Park, **(B)** Lake Agawam, **(C)** Lake Erie, **(D)** Honeoye Lake, and **(E)** Lake Neatahwanta, including nitrate, ammonium, urea, and glutamic acid. Systems had highest rates in free-living fraction, excluding **(B)** Lake Agawam, which had a high relative abundance of *Pseudanabaena*, and **(E)** Lake Neatahwanta, which had similar PON concentrations in both free-living and *Microcsytis* colony fractions. Systems had the highest uptake of urea (*p* < 0.05), excluding **(A)** the Lake in Central Park and **(B)** Lake Agawam where ammonium had higher uptake (*p* < 0.01). Letter denotations indicate significance between water groups.

**Figure 7:**
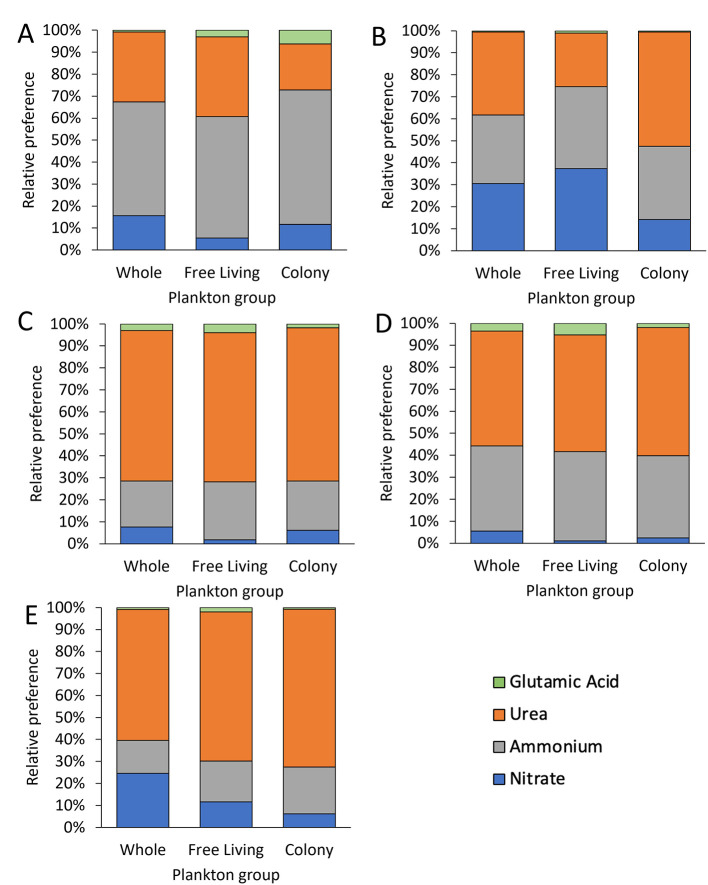
Mean relative preference for nitrogen uptake using volumetric nitrogen uptake of whole water and fractions for **(A)** the Lake in Central Park, **(B)** Lake Agawam, **(C)** Lake Erie, **(D)** Honeoye Lake, and **(E)** Lake Neatahwanta, including nitrate, ammonium, urea, and glutamic acid. All systems had highest preference for urea (*p* < 0.05), excluding **(A)** the Lake in Central Park and **(B)** Lake Agawam where ammonium had higher uptake (*p* < 0.05).

In the Lake in Central Park, volumetric N uptake rates and N specific uptake rates differed by plankton group (*p* < 0.001 for both) and by N source (*p* < 0.001 for both; Two-way ANOVA), while N preference differed by N source only (*p* < 0.001; Two-way ANOVA). *Microcystis* colony volumetric uptake rates were significantly greater than the free-living fraction (*p* < 0.05; Tukey; Figure 5A, Supplementary Figure 8). In addition, ammonium uptake rates exceeded rates for nitrate and glutamic acid for all plankton groups (*p* < 0.05; Tukey; Table 2, Figure 5A, Supplementary Figure 8). N specific uptake by the free-living fraction was significantly greater than both whole water and the *Microcystis* colony fraction (*p* < 0.001 for both; Tukey; Table 3, Figure 6A). Specifically, ammonium specific uptake was significantly higher within the free-living fraction compared to the whole water and *Microcystis* colony fraction (*p* < 0.001; Tukey) with no difference between the whole water and *Microcystis* colony fraction. In addition, ammonium specific uptake within the free-living fraction was significantly greater than both nitrate and glutamic acid uptake (*p* < 0.001 and *p* = 0.001, respectively; Tukey; Table 3, Figure 6A). N preference for ammonium and urea did not differ from each other but were significantly greater than nitrate and glutamic acid (*p* < 0.05; Tukey). All plankton groups had greatest preference for ammonium at 52% for whole water, 55% for free-living fraction, and 61% for *Microcystis* colony fraction (Figure 7A). While communities preferentially assimilated ammonium on average across the year, urea was often the preferred form in summer months (Figure 7A; Supplementary Figure 9).

**Table 2:** Nitrogen volumetric uptake rates of whole water (WW) and fractions (free-living, FL; *Microcystis* colony, MC) during the Lake Agawam (LA) time series, the Lake in Central Park time series (LCP), the Lake Erie (LE) transects (sites M1–M2), and individual sampling sites for Honeoye Lake (HE) and Lake Neatahwanta (NT).

Lake	Date	Plankton group	Rate (μmol L^−1^ h^−1^)				Lake	Date	Plankton group	Rate (μmol L^−1^ h^−1^)			
			Nitrate	Ammonium	Urea	Glutamic acid				Nitrate	Ammonium	Urea	Glutamic acid
LA	6/17/20	WW	12.1	4.31	2.17	0.010	LCP	7/21/21	WW	0.17	0.31	1.73	0.028
		FL	2.94	1.74	0.92	0.013			FL	0.02	0.08	0.39	0.014
		MC	0.99	3.10	1.13	0.059			MC	0.01	0.15	0.84	0.01
LA	7/2/20	WW	0.31	0.39	1.72	0.026	LCP	8/4/21	WW	0.21	0.44	4.18	0.055
		FL	0.00	0.05	0.06	0.005			FL	0.01	0.03	0.19	0.007
		MC	0.52	1.68	6.61	0.096			MC	0.22	0.46	4.15	0.061
LA	7/15/20	WW	0.71	0.63	1.28	0.021	LCP	10/14/21	WW	2.01	0.88	0.89	0.050
		FL	0.01	0.07	0.06	0.009			FL	0.01	0.02	0.02	0.007
		MC	0.76	0.65	1.05	0.014			MC	0.91	0.68	–	2.96
LA	7/29/20	WW	0.73	0.35	1.46	0.012	LCP	7/27/22	WW	1.62	1.75	0.75	0.042
		FL	0.01	0.08	0.06	0.005			FL	0.05	0.4	0.07	0.02
		MC	1.16	0.67	2.48	0.015			MC	1.61	5.61	0.68	0.027
LA	8/13/20	WW	0.28	0.65	1.74	0.011	LCP	8/11/22	WW	0.35	7.60	1.45	0.081
		FL	0.03	0.15	0.27	0.003			FL	0.02	0.47	0.09	0.011
		MC	0.47	1.18	3.00	0.023			MC	0.02	6.93	–	–
LA	8/26/20	WW	0.84	0.81	2.59	0.011	LCP	9/2/22	WW	1.38	7.95	2.61	0.057
		FL	0.01	0.10	0.19	0.004			FL	0.01	0.25	0.06	0.011
		MC	0.65	1.12	3.68	0.014			MC	3.13	17.2	4.89	0.14
LA	9/9/20	WW	0.66	0.51	1.78	0.014	LE M1	8/19/21	WW	0.33	0.18	1.11	0.124
		FL	0.00	0.02	0.04	0.002			FL	0.04	0.08	0.23	0.055
		MC	2.10	1.63	6.41	0.046			MC	0.17	0.14	0.65	0.035
LA	9/21/20	WW	0.70	0.48	0.55	0.016	LE M2	8/18/22	WW	0.56	1.71	6.14	0.185
		FL	0.21	0.07	0.02	0.004			FL	0.07	1.06	2.98	0.140
		MC	0.00	0.78	1.22	0.017			MC	0.29	1.67	5.67	0.125
LA	10/8/20	WW	0.81	0.41	–	0.016	LE M2	8/19/22	WW	0.12	2.30	5.65	0.234
		FL	0.01	0.06	–	0.004			FL	0.02	1.58	3.65	0.140
		MC	1.02	0.53	–	0.013			MC	0.09	2.68	7.05	0.184
LA	10/19/20	WW	1.53	0.31	1.48	0.014	HE	9/16/21	WW	0.08	0.54	0.72	0.048
		FL	0.02	0.02	0.04	0.003			FL	0.01	0.23	0.30	0.029
		MC	0.78	0.47	0.60	0.016			MC	0.04	0.58	0.90	0.030
LA	10/27/20	WW	0.53	0.33	1.82	–	NT	9/17/21	WW	0.71	0.43	1.71	0.027
		FL	0.01	0.03	0.07	–			FL	0.08	0.12	0.45	0.014
		MC	0.41	0.53	2.94	–			MC	0.14	0.49	1.65	0.018
LA	11/17/20	WW	0.02	0.33	0.30	–							
		FL	0.01	0.02	0.02	–							
		MC	0.02	0.34	0.12	–							
LA	7/14/21	WW	0.26	0.32	1.48	0.031							
		FL	0.01	0.02	0.04	0.005							
		MC	0.29	0.37	1.71	0.032							
LA	7/28/21	WW	0.29	0.28	1.25	0.028							
		FL	0.01	0.03	0.03	0.007							
		MC	0.10	0.31	1.39	0.021							
LA	8/2/22	WW	1.74	11.3	5.83	0.083							
		FL	0.03	0.54	0.14	0.009							
		MC	1.80	11.4	6.53	0.098							
LA	9/7/22	WW	3.58	4.16	3.69	0.097							
		FL	0.03	0.34	0.10	0.025							
		MC	1.22	4.21	3.45	0.054							

**Table 3:** Nitrogen specific uptake rates of whole water (WW) and fractions (free-living, FL; *Microcystis* colony, MC) during the Lake Agawam (LA) time series, the Lake in Central Park time series (LCP), the Lake Erie (LE) transects (sites M1–M2), and individual sampling sites for Honeoye Lake (HE) and Lake Neatahwanta (NT).

Lake	Date	Plankton group	Rate (d^−1^)				Lake	Date	Plankton group	Rate (d^−1^)			
			Nitrate	Ammonium	Urea	Glutamic acid				Nitrate	Ammonium	Urea	Glutamic acid
LA	6/17/20	WW	–	–	–	–	LCP	7/21/21	WW	0.0297	0.0538	0.306	0.0049
		FL	–	–	–	–			FL	0.0037	0.0767	0.436	0.0052
		MC	–	–	–	–			MC	0.0062	0.0200	0.0994	0.0035
LA	7/2/20	WW	0.0071	0.0091	0.0396	0.00060	LCP	8/4/21	WW	0.0035	0.0073	0.0703	0.0009
		FL	0.0005	0.0061	0.0073	0.0006			FL	0.0001	0.00046	0.0033	0.00012
		MC	0.0127	0.0407	0.160	0.0023			MC	0.0499	0.106	0.953	0.014
LA	7/15/20	WW	0.0196	0.0174	0.0355	0.00059	LCP	10/14/21	WW	0.0309	0.0135	0.0137	0.0008
		FL	0.0012	0.0075	0.0060	0.0009			FL	0.0002	0.00027	0.00028	0.00012
		MC	0.0249	0.0215	0.0347	0.0005			MC	0.451	0.336	–	1.47
LA	7/29/20	WW	0.102	0.0487	0.203	0.00165	LCP	7/27/22	WW	0.113	0.122	0.0524	0.0029
		FL	0.0136	0.0816	0.0639	0.0051			FL	0.00482	0.0377	0.00626	0.0019
		MC	0.203	0.118	0.4341	0.0027			MC	0.802	2.78	0.337	0.0132
LA	8/13/20	WW	0.0145	0.0338	0.0902	0.00055	LCP	8/11/22	WW	0.0116	0.255	0.0487	0.0027
		FL	0.0276	0.155	0.282	0.0033			FL	0.00087	0.0251	0.0049	0.0006
		MC	0.0232	0.0579	0.147	0.0011			MC	0.0057	2.26	–	–
LA	8/26/20	WW	0.163	0.156	0.502	0.0021	LCP	9/2/22	WW	0.0491	0.282	0.0925	0.0020
		FL	0.0118	0.0932	0.177	0.0032			FL	0.00051	0.00866	0.00206	0.00037
		MC	0.139	0.238	0.78	0.0029			MC	0.625	3.44	0.977	0.028
LA	9/9/20	WW	0.0178	0.0137	0.0481	0.00039	LE M1	8/19/21	WW	0.0366	0.0203	0.124	0.0139
		FL	0.0021	0.0130	0.0242	0.0013			FL	0.0492	0.103	0.303	0.0712
		MC	0.0793	0.0616	0.242	0.0017			MC	0.0347	0.030	0.135	0.0072
LA	9/21/20	WW	0.100	0.0693	0.0799	0.0023	LE M2	8/18/22	WW	0.122	0.373	1.34	0.0402
		FL	0.125	0.0392	0.0120	0.0025			FL	0.0662	1.02	2.89	0.135
		MC	0.0007	0.167	0.261	0.0037			MC	0.119	0.676	2.30	0.0505
LA	10/8/20	WW	0.080	0.0407	–	0.0016	LE M2	8/19/22	WW	0.0978	0.0521	0.321	0.0154
		FL	0.0069	0.0451	–	0.0034			FL	0.0244	0.0195	0.124	0.0390
		MC	0.1208	0.0628	–	0.0015			MC	0.0166	0.0118	0.0569	0.0014
			**Nitrate**	**Ammonium**	**Urea**	**Glutamic acid**				**Nitrate**	**Ammonium**	**Urea**	**Glutamic acid**
LA	10/19/20	WW	0.185	0.0379	0.180	0.0017	HE	9/16/21	WW	0.0089	0.063	0.0852	0.0057
		FL	0.0156	0.0165	0.0279	0.0020			FL	0.0053	0.0829	0.129	0.0043
		MC	0.0892	0.0538	0.0690	0.0018			MC	0.0075	0.296	0.386	0.0383
LA	10/27/20	WW	0.0511	0.0314	0.174	–	NT	9/17/21	WW	0.106	0.0645	0.256	0.0040
		FL	0.0045	0.0153	0.0354	–			FL	0.0262	0.0418	0.153	0.0046
		MC	0.0475	0.0611	0.3410	–			MC	0.0455	0.157	0.531	0.0056
LA	11/17/20	WW	0.0019	0.0401	0.0363	–							
		FL	0.0034	0.0091	0.0113	–							
		MC	0.0026	0.0531	0.0193	–							
LA	7/14/21	WW	0.0069	0.0086	0.0396	0.00083							
		FL	0.0044	0.0174	0.0345	0.0043							
		MC	0.0086	0.0111	0.0515	0.0010							
LA	7/28/21	WW	0.0521	0.0505	0.223	0.0050							
		FL	0.0040	0.0221	0.0170	0.0049							
		MC	0.0221	0.0693	0.31	0.0046							
LA	8/2/22	WW	0.0453	0.293	0.152	0.0021							
		FL	0.0264	0.420	0.110	0.0068							
		MC	0.0469	0.295	0.170	0.0025							
LA	9/7/22	WW	0.578	0.672	0.596	0.0156							
		FL	0.0396	0.463	0.132	0.0342							
		MC	0.238	0.822	0.674	0.0106							

In Lake Agawam, volumetric N uptake rates and N-specific uptake rates differed by N source (*p* < 0.001, *p* < 0.05, respectively) and by plankton group (*p* < 0.001, *p* < 0.05, respectively) while relative preference differed by N source only (*p* < 0.05; Two-way ANOVA, Tukey). Ammonium and urea volumetric uptake rates were significantly greater than glutamic acid uptake rates for all plankton groups (*p* < 0.05 for all; Tukey) and urea was assimilated fastest by the whole water and *Microcystis* colony fraction (*p* < 0.05 for all; Tukey; Table 2, Figure 5B, Supplementary Figure 10). *Microcystis* colony fraction and whole water volumetric uptake rates were significantly greater than free-living fraction (*p* < 0.001; Tukey; Figure 5B, Supplementary Figure 10) while N-specific colony fraction uptake rates were significantly greater than the free-living fraction (*p* < 0.05; Tukey; Table 3,Figure 6B). Specifically, urea specific uptake was significantly greater than nitrate (*p* < 0.001; Tukey) and significantly higher within the colony fraction compared to the free-living fraction and whole water (*p* = 0.003) with no difference between the free-living fraction and whole water (Figure 6B). N-specific uptake of urea was also significantly greater than glutamic acid and nitrate within the *Microcystis* colony fraction (*p* < 0.001; Tukey; Table 3, Figure 6B). N preference was significantly different between all source comparisons and within each plankton group, except for nitrate compared to ammonium in whole water and ammonium compared to urea in the free-living fraction, while nutrient preference by plankton group was only significantly different between the whole water and free-living fraction for nitrate and ammonium (*p* < 0.05; Tukey). Lake Agawam plankton groups had different relative preferences for N between groups: Specifically urea was highest in the whole water and *Microcystis* colony fraction (38% and 52%, respectively) while nitrate and ammonium were evenly preferred in the free-living fraction (37% for both; Figure 7B). Throughout the time-series, there were seasonal changes in N preferences, with nitrate preferred in spring and fall and urea preferred in summer, contributing to higher relative preferences for nitrate in this system compared to other systems (30% whole Supplementary Figure 11).

In Lake Erie, volumetric N uptake rates, N-specific uptake rates, and relative preference differed by N source (*p* < 0.001, *p* < 0.001, and *p* < 0.005, respectively) but not by plankton group (*p* > 0.05; Two-way ANOVA; Figures 5C, 6C, 7C). Volumetric uptake rates of urea were significantly higher than all other N sources for all plankton groups (*p* < 0.05 for all; Tukey; Table 2, Figure 5C, Supplementary Figure 12). N-specific uptake for urea was also higher than nitrate and glutamic acid within the free-living fraction (*p* = 0.002 for both; Tukey; Table 3, Figure 6C). Similarly, relative preferences for urea (69%, 68%, and 71% for whole water, free-living fraction, and *Microcystis* colony fraction, respectively) were significantly greater than all other N sources (*p* < 0.001 for all; Tukey). Additionally, N preference was significantly different between all N sources (*p* < 0.05; Tukey) except for ammonium compared to nitrate and nitrate compared to glutamic acid (Figure 7C, Supplementary Figure 13).

In Honeoye Lake, volumetric N uptake rates and relative preference differed by N source (*p* < 0.05 for both; Tukey test) but not by plankton group (*p* > 0.05; Two-way ANOVA; Figures 5D, 7D), while N-specific uptake rates did not differ by N source or by plankton group (*p* = 0.061 and *p* = 0.088, respectively; Two-way ANOVA; Table 3, Figure 6D). Urea volumetric uptake rates were significantly higher than nitrate and glutamic acid rates (*p* < 0.05 for both; Table 2, Figure 5D). Relative preferences for urea (52%, 53%, and 58% for whole water, free-living fraction, and *Microcystis* colony fraction, respectively) were significantly greater than all other sources (*p* < 0.05 for all; Tukey), while preference for ammonium (39%, 41%, and 38%, respectively) was significantly greater than nitrate and glutamic acid (*p* < 0.001 for both; Tukey) which did not differ from each other.

Finally, in Lake Neatahwanta, volumetric N uptake, N-specific uptake, and relative preference rates all differed by N source (*p* < 0.05 for all; Tukey) but not by plankton group (*p* > 0.05; Two-way ANOVA; Figures 5E, 7E). Volumetric urea uptake rates were significantly higher than nitrate and glutamic acid rates (*p* < 0.05 for all; Tukey; Table 2; Figure 5E). Similarly, N-specific urea uptake was significantly higher than glutamic acid (*p* < 0.05 for both; Tukey; Table 3, Figure 6E). Relative preferences for urea (59%, 68%, and 72% for whole water, free-living fraction, and *Microcystis* colony fraction, respectively) were significantly greater than all other sources (*p* < 0.05 for all; Tukey), while preference for ammonium (15%, 19%, and 21%, respectively) was significantly greater than glutamic acid (*p* < 0.05 for both; Tukey) although there was no significant difference between ammonium and nitrate.

### Relationships between N assimilation and environmental conditions

**Figure 8:**
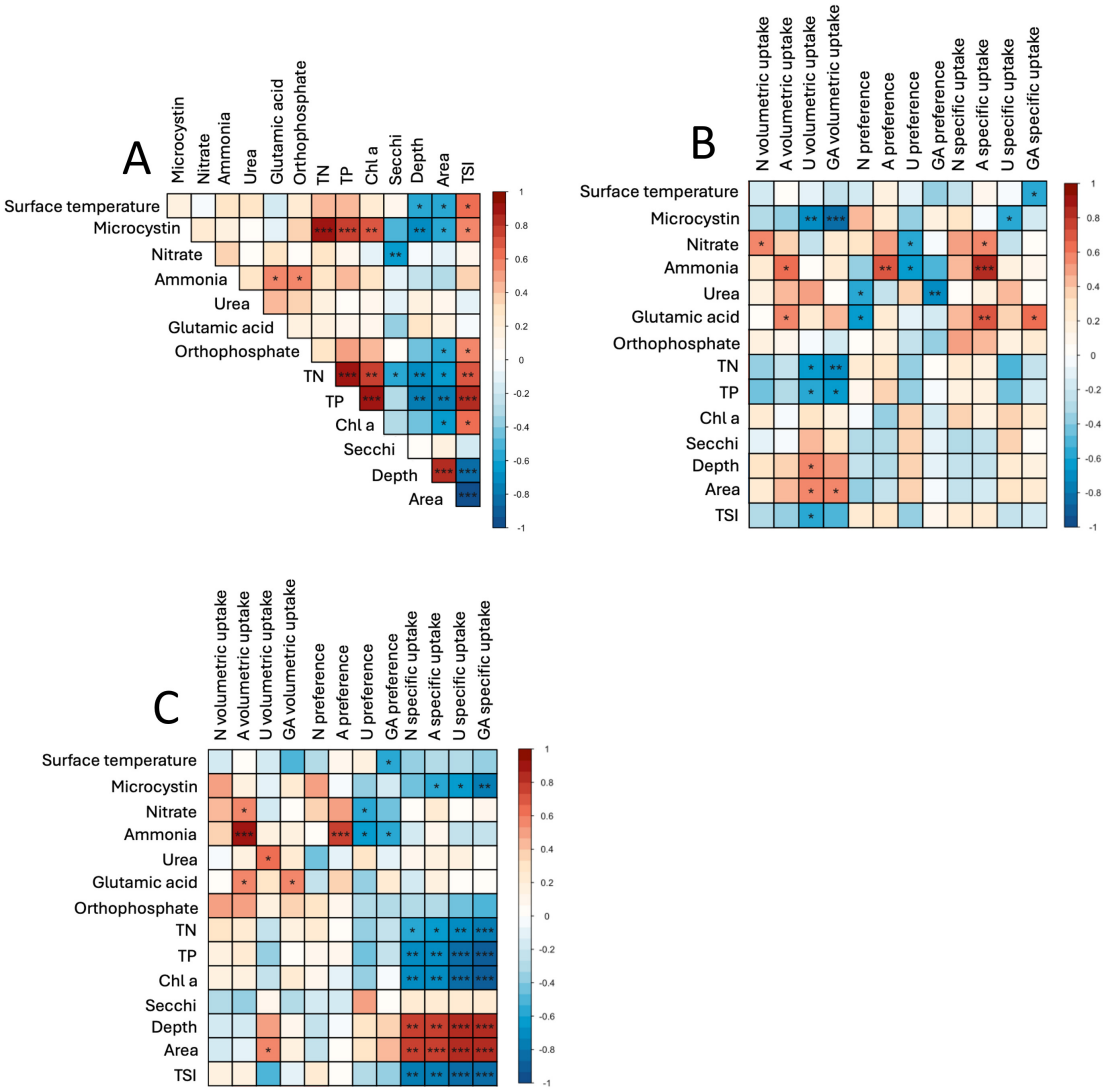
Correlation matrices conducted for **(A)** whole water, **(B)** free-living fraction, and **(C)** *Microcystis* colony fraction for 2021 and 2022, including relationships between environmental data, volumetric uptake rates, N-specific uptake rates, and preference. Gradient indicates direct (red) and indirect (blue) correlations and ranges from −1.0 to 1.0; Asterisks represent significance at *p < 0.05, **p < 0.01, and ***p < 0.001.

Across all sites and years, multiple statistical associations were found between N-uptake rates and environmental conditions (Figure 8; Supplementary Figure 14). All N-specific uptake rates within the *Microcystis* colony fraction were significantly and inversely correlated with chl-*a*, TN, TP, and microcystin (*p* < 0.05 for all), excluding nitrate specific uptake and microcystin (Figure 8C). TN, TP, chl-*a*, and microcystin were also significantly and inversely correlated with N-specific urea and glutamic acid uptake in whole water (*p* < 0.05; Supplementary Figure 14A). Volumetric uptake rates of urea and glutamic acid within the free-living fraction were also significantly and inversely correlated with microcystin, TN, and TP (*p* < 0.05; Figure 8B; Supplementary Figure 14B). Lake depth was significantly correlated with volumetric urea uptake within the free-living fraction (*p* < 0.05) and all N-specific uptake within the *Microcystis* colony fraction (*p* < 0.05; Figures 8A–C; Supplementary Figures 14A–C). Additionally, temperature, microcystin, TN, and TP were significantly and inversely correlated with lake area, lake depth, total chl-*a* and chl-*a* of *Microcystis* colonies (*p* < 0.05 for all; Figure 8A; Supplementary Figure 14C). Lake trophic state index (TSI) was significantly correlated with microcystin and surface temperature (*p* < 0.05) and significantly and inversely correlated with lake area and lake depth (*p* < 0.001; Figure 8A). Microcystin was also significantly correlated with TN and TP (*p* < 0.01) and chl-*a* in the whole water and the *Microcystis* colony fraction (Figure 8A; Supplementary Figures 14A, C).

### N assimilation relative to microbial compositions

**Figure 9:**
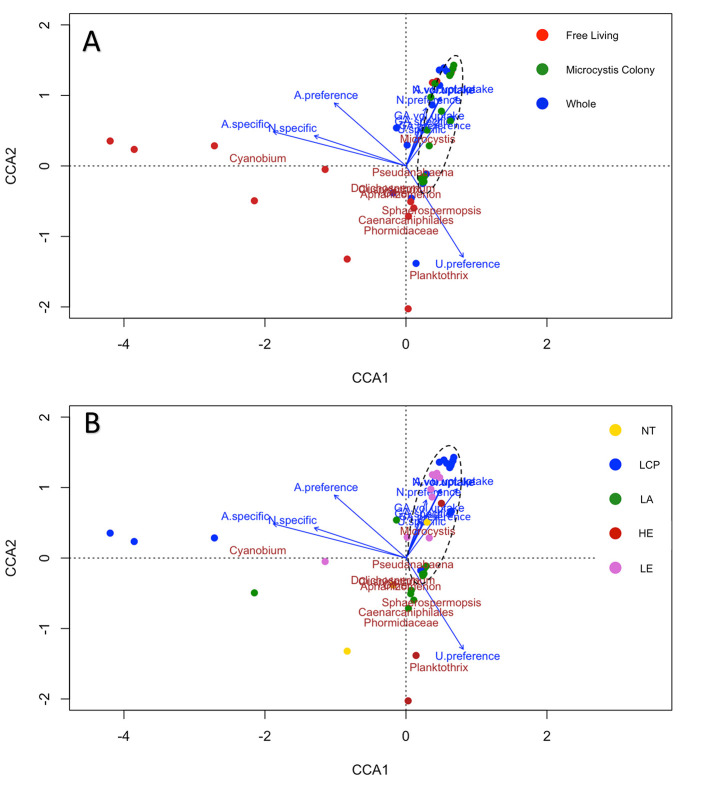
Canonical correlation analysis (CCA) showing strength (length) and correlation (direction) of uptake rates of interest (bi-plot arrows) to the 16S community clusters for cyanobacterial abundances, colors denote **(A)** whole water and fractions, and **(B)** lake. Circles indicate clustering of *Microcystis* colony-associated communities. N, A, and U volumetric uptake overlap at the top, U and GA specific, GA volumetric, and GA preference overlap in the center of the *Microcystis* colony-associated communities. Proportion explained CCA1: 44.85%, CCA2: 25.72%.

Cyanobacterial diversity was compared to N assimilation rates by the plankton groups using canonical correlation analysis (CCA). Cyanobacterial community composition explained 71% of the variance of N uptake rates between dimension one (45%) and dimension two (26%; Figure 9). CCA highlighted a clustering of *Microcystis* colony fraction and whole water samples separate from free-living fraction samples (Figure 9A). The whole water and the *Microcystis* colony fraction clustered in close association with *Microcystis*, and, to a lesser extent, *Pseudanabaena*, while the free-living fraction was dispersed and associated with other cyanobacterial genera (Figure 9A). The *Microcystis* colony fraction most associated with *Pseudanabaena* were from Lake Agawam (Figure 9B). Most volumetric and specific N uptake rates and preferences were most closely associated with whole water and *Microcystis* colony fraction and *Microcystis* (Figure 9). The exceptions were ammonium and nitrate specific uptake which was more associated with the free-living fraction; urea and ammonium preference were equally associated with the free-living and colony fractions (Figure 9). A similar clustering of uptake rates was observed within the total microbial community (Supplementary Figure 15A) and among the heterotrophic bacterial communities (Supplementary Figure 15B).

## Discussion

This study characterized the composition and concentration of *Microcystis* and associated microbes via high throughput sequencing of 16S rRNA while quantifying N assimilation by *Microcystis* colonies and other co-occurring plankton. Plankton fractionation revealed the largest differences in bacterial and cyanobacterial community composition between years and across ecosystems as the *Microcystis* colony fraction was significantly different from the other plankton groups. N uptake rates varied by N forms and, in some systems, differed by plankton groups, with urea assimilating at the highest rates in most systems and across groups. N uptake patterns were closely linked with the trophic status of lakes (TN, TP, chl-*a*) as well as with lake depth and lake area. Collectively, these findings provide insight regarding the differences in, and drivers of, N-assimilation by plankton communities during *Microcystis* blooms.

### Microbial community composition

This study found differences in heterotrophic bacterial diversity between free-living assemblages and *Microcystis*-associated assemblages that persisted across large spatial scales, a finding consistent with prior studies (Akins et al., 2018; Cook et al., 2020; Hoke et al., 2021; Jankowiak and Gobler, 2020; Smith et al., 2021). This was also the case for cyanobacterial assemblages, with *Microcystis* comprising the majority (66–84%) of sequences within the colony fraction for four of five systems. Lake Agawam was the exception as all groups in this system contained high relative abundances of *Pseudanabaena* (≥50% for all fractions), despite the *Microcystis* colony fraction macroscopically appearing similar to other systems. Microscopically, small (~2μm), rod-like plankton were found within the phycosphere of *Microcystis* colonies within Lake Agawam that were not present in other systems (Supplementary Figure 16B). These findings are consistent with the small size (2 μm) of *Pseudanabaena* cells and prior analyses of Lake Agawam colonies (Jankowiak and Gobler, 2020). *Pseudanabaena* is a cryptic, picoplankton (Zwart et al., 2005; Willame et al., 2006; Acinas et al., 2009) that has been previously molecularly identified within large *Microcystis* blooms (Berry et al., 2017; Jankowiak et al., 2019; Jankowiak and Gobler, 2020) and in other freshwater and brackish systems (Zwart et al., 2005; Willame et al., 2006; Tuji and Niiyama, 2018). Despite the high relative abundance of *Pseudanabaena* in Lake Agawam, the biovolume of these 2 μm rods is likely 50-fold less than *Microcystis* cells, which are generally 5 μm spheres, meaning that *Microcystis* remained the dominating taxon for cyanobacterial biomass in Lake Agawam as well.

Moreover, the relative frequency of cyanobacteria based on sequencing of 16S rRNA may also be influenced by genome copy number which can vary across cyanobacterial genera (Schirrmeister et al., 2012). Regardless, *Microcystis* colonies within the colony fraction harbored and supported bacterial communities that were compositionally distinct from the free-living fraction, emphasizing the role of colonies as structured microenvironments.

### N uptake by nutrient source

Differences in nitrogen assimilation were strongly driven by N source within this study for volumetric uptake, N-specific uptake, and N preference. In the three large lakes (Lake Erie, Honeoye Lake, and Lake Neatahwanta), communities preferentially assimilated urea across all plankton groups. For Lake Agawam communities, there were seasonal changes in N preference but, on average, the whole water and *Microcystis* colony fraction displayed a preference for urea while the free-living fraction preferred nitrate and ammonium. For the Lake in Central Park, all plankton groups preferentially assimilated ammonium, although urea was frequently the preferred form during summer months. Within these systems, urea concentrations remained relatively consistent during summer compared to nitrate and ammonium, which were depleted, suggesting internal regeneration of urea at this time. Plankton and cyanobacterial communities often preferentially assimilate ammonium due to its lower energy requirements for cellular use (McCarthy et al., 1977; Herrero et al., 2001; Dugdale et al., 2007; Paerl et al., 2011). However, the preference for urea within all lake systems during summer months supports recent findings identifying urea as a key nutrient that can support and promote CHABs (Belisle et al., 2016; Chaffin and Bridgeman, 2014; Krausfeldt et al., 2019; Newell et al., 2019). This is also consistent with studies suggesting that urea can promote production by cyanobacteria and other prokaryotes (Berg et al., 2001; Glibert et al., 2004, 2008). Urea can be regenerated by heterotrophic bacteria (Berg and Jørgensen, 2006; Solomon et al., 2010), zooplankton (Miller and Roman, 2008; Painter et al., 2008; Solomon et al., 2010), or sediment (Lomstein et al., 1989; Antia et al., 1991; Solomon et al., 2010), or can be derived from urea-based fertilizers which have globally increased in use over 100-fold since the 1960s (Glibert et al., 2006, 2008; Paerl et al., 2016). Urea-based fertilizers have become the predominant chosen N-fertilizer in the United States at ~90% of N-fertilizers used as of 2011 at over 5 million tons consumed per year (Paerl et al., 2016). Although urea has become prominent in agriculture since the 1960s, it is rarely measured in water quality analyses even with links of urea to increased primary production (Belisle et al., 2016; Paerl et al., 2016). Additionally, urease, the enzyme responsible for cleaving urea molecules into ammonia ions, is not repressed by other nitrogen sources, including ammonium, in cyanobacteria (Herrero et al., 2001). Overall, urea enrichment has been proposed to alter the composition of planktonic species and lead to the development and increase of harmful algal blooms (Glibert et al., 2008), highlighting the importance in considering this compound as a driver of CHABs (Belisle et al., 2016). These results reinforce the need to consider urea alongside inorganic nitrogen forms when evaluating drivers of bloom development and persistence.

The second most preferred compound was often ammonium, with nitrate preference being higher in spring in systems where seasonal measurements were made, similar to previous studies (McCarthy et al., 1977; Bronk et al., 1998; Moschonas et al., 2017; Andersen et al., 2020). Nitrate is known to be utilized during spring and depleted by summer due to increases in planktonic biomass and uptake rates associated with warmer temperatures (Berg et al., 2003; Gobler et al., 2016; Moschonas et al., 2017). This forces plankton communities to rely on regenerated N sources, such as urea and ammonium, during summer months (Bronk and Ward, 1999; Hasegawa et al., 2001). N regeneration can play an important role in sustaining CHABs compared to external inputs (Paerl et al., 2015; Newell et al., 2019; Hoffman et al., 2022) particularly within eutrophic lakes, including Lake Taihu (Hampel et al., 2018; Jiang et al., 2019), Lake Champlain (McCarthy et al., 2013), and Lake Erie (Hoffman et al., 2022; Kharbush et al., 2023). Berg et al. (2003) also found that uptake of regenerated N in the form of ammonium, urea, and other amino acids was important for sustaining cyanobacteria and other planktonic species, which was also seen here. During summer months when nitrate was depleted within Lake Agawam and the Lake in Central Park, urea became the preferred N form. While ammonium is traditionally considered the primary form of regenerated N supporting CHABs, this study demonstrates that urea was more important for supporting CHABs dominated by *Microcystis*. Therefore, the management and reduction of urea from anthropogenic sources may be fundamental to controlling and reducing the prevalence of CHABs.

### N uptake by fraction

Nitrogen uptake rates, both volumetric and N-specific, differed consistently between plankton groups while relative nitrogen preferences were similar. The free-living fraction exhibited lower volumetric uptake rates than both the whole water and *Microcystis* colony fraction, with significant differences found for Lake Agawam and the Lake in Central Park. This outcome was consistent with the large biomass differences across fractions with levels of cyanobacterial biomass and PON being consistently lower (1-to-200-fold and 1-to-30-fold, respectively) in the free-living fraction. When uptake was normalized to PON (specific uptake), however, the free-living fraction frequently displayed higher uptake rates than the whole water and *Microcystis* colony fraction, an outcome likely a function of size-dependent physiological advantages, specifically surface area-to-volume ratios, allowing for assimilation of dissolved constituents at faster rates (Beveridge, 1988; Koch, 1996; Schulz and Jørgensen, 2001; Young, 2006). While this was the case for Lake in Central Park, Lake Erie, and Honeoye Lake, the *Microcystis* colony fraction maintained the highest N-specific uptake rates in Lake Agawam and Lake Neatahwanta. Lake Agawam was the only ecosystem studied where *Pseudanabaena* comprised >50% of the 16S relative frequency in all fractions, potentially accounting for the free-living fraction not dominating specific uptake rates. Lake Neatahwanta was the only ecosystem studied where the cyanobacterial biomass and PON concentrations were similar for both the free-living and *Microcystis* colony fractions, accounting for both the volumetric and specific uptake having similar trends.

### N uptake by lake characteristics

N-uptake rates were related to environmental characteristics encapsulated by the Trophic State Index (TSI), a numerical system that uses environmental parameters, including TP and chl-*a*, to rank ecosystems as hypereutrophic (>74), eutrophic (54–74), mesotrophic (44–54), oligotrophic (24–44), and ultraoligotrophic (< 24; Carlson, 1977; Toledo et al., 1983; Toledo and Companhia de Tecnologia de Saneamento Ambiental (CETESB), 1990; Paulic et al., 1996; Klippel et al., 2020). Here, the Lake in Central Park and Lake Agawam were hypereutrophic systems (93 and 79, respectively) while Honeoye Lake, Lake Erie, and Lake Neatahwanta ranked as eutrophic (73, 69, and 68, respectively). Overall, TSI was inversely correlated with depth and area, and directly correlated with surface temperature, microcystin, chl-*a*, TN, and TP levels across all systems within whole lake water (Figure 8A), indicating higher productivity within the smaller, hypereutrophic systems. Specifically, the Lake in Central Park and Lake Agawam are the smallest (0.5 km^2^) and shallowest (2 and 3 m, respectively) lakes studied and had the highest TSI, with the highest temperatures and highest levels of TN, TP, and chl-*a*. In contrast, Honeoye Lake and Lake Neatahwanta were over 100-fold larger (7.2 and 3.1 km^2^, respectively) and 2-to-4-fold deeper (9 and 4 m, respectively), with the lowest TSI levels and the lowest temperatures and levels for TN, TP, and chl-*a*. Connecting these parameters to N uptake rates, the shallower, hypereutrophic systems harbored the highest *Microcystis* colony fraction volumetric N uptake rates but the lowest *Microcystis* colony fraction specific N uptake rates, while the deeper and less eutrophied systems had lower *Microcystis* colony fraction volumetric N uptake rates but the highest *Microcystis* colony fraction N specific uptake rates. Lake area and nutrient loading typically drive trophic states as small lakes with high catchment area-to-lake volume ratios generally have a more limited capacity to dilute and assimilate nutrients compared to larger systems, resulting in a greater sensitivity to excessive nutrient loads (Tammelin and Kauppila, 2018; Zhou et al., 2022). Such systems are more likely to be eutrophic and experience CHABs, especially in urban areas (Moore et al., 2003; Naselli-Flores, 2008; Qin et al., 2006; Zhou et al., 2022), and to be strongly influenced by sedimentary nutrient fluxes (Havens and James, 2005; Zhou et al., 2022). TN, TP, and chl-*a* are also well-known proxies for high biomass CHABs (Beaulieu et al., 2013; Siegel et al., 2013) with TN and temperature shown to explain the most variance for cyanobacterial biomass across US lakes (Beaulieu et al., 2013).

Differences in N assimilation were related to multiple environmental and physical characteristics of each lake study. Across all systems, N-specific uptake rates of all N sources by the *Microcystis* colony fraction were significantly and inversely correlated with TN, TP, chl-*a*, TSI, and microcystin and were significantly and directly correlated with lake area and lake depth. Similarly, the volumetric uptake rates of urea and glutamic acid by the free-living fraction were inversely and significantly correlated with TN, TP, microcystin, and TSI, while significantly and directly correlated with lake area and lake depth, excluding volumetric glutamic acid and depth. Eutrophic lakes generally host larger, denser CHABs (Chislock et al., 2013; Davidson et al., 2014; Wurtsbaugh et al., 2019), yet the volumetric N uptake rates of the free-living fraction were negatively correlated with symptoms of denser blooms (TN, TP, chl-*a*) as were N-specific uptake rates by the colony fraction. These inverse correlations, therefore, indicate that as blooms intensified, free-living plankton took up less N and *Microcsytis* colonies and its associated plankton took up less N when normalized to the total amount of particulate N. While potentially seeming paradoxical, these findings provide insight into the dynamics of N assimilation during *Microcystis* blooms. Regarding free-living plankton, this trend may reflect *Microcystis* and associated plankton displaying progressively larger volumetric N uptake rates as blooms intensified, resulting in greater competition for the existing N pools (Tubay et al., 2013) and thus lower volumetric uptake rates by free-living plankton. Regarding colonies, as blooms progress, *Microcystis* colonies increase in size and decrease in compactness, with an increasingly loose cell arrangement (Xiao et al., 2018; Xu et al., 2023; Feng et al., 2020, 2024). This morphological change increases the distance of cells within the polysaccharide envelope from the external environment, extending the physical distance nutrients must diffuse for uptake to occur. This physical distance can be seen in the difference in *Microcystis* colonies between the sites within this study, with colonies in hypereutrophic systems being larger and having a looser arrangement of cells within colonies (Supplementary Figures 16A, B) compared to a more compact arrangement in the eutrophic systems (Supplementary Figures 16C–E). Following Fick's first law of diffusion, an increase in the size of colonies would reduce the rate of N uptake by *Microcystis* cells in colonies (Philibert, 2006; McMurtrey, 2016). In addition, as nitrogenous nutrients traverse the polysaccharide layer, plankton hosted within this envelope (Kuentzel, 1969; Hoppe, 1981; Worm and Søndergaard, 1998; Parveen et al., 2013; Jankowiak and Gobler, 2020) will compete for these essential nutrients (Li et al., 2024; Seymour et al., 2017). It is important to note that other factors could also influence these correlations, including allelopathy and community differences between sites. Specifically, cyanobacteria can produce allelopathically active compounds that may limit productivity of surrounding organisms from available resources (Gross, 2003; Chia et al., 2018; Hernández-Zamora et al., 2021). Collectively, this indicates a complex relationship between lake trophic state and N uptake rates that is also likely influenced by interspecies competition and the physics of colony-associated micro-environments.

### Influence of microbial community composition on N uptake

Across all lakes studied, whole water and the *Microcystis* colony fraction were associated with the genera *Microcystis* and, to a lesser extent, *Pseudanabaena* while the free-living fraction associated with all other prominent cyanobacteria such as *Cyanobium, Planktothrix, Aphanizomenon*, and *Dolichospermum*. CCA revealed a tight clustering of most N uptake rates with the relative abundance of *Microcystis*, excluding ammonium and nitrate specific uptake and ammonium preference, which were each more associated with free-living fraction samples and other cyanobacterial genera. This finding suggests that *Microcystis* and associated microbes within colonies controlled the volumetric assimilation of all N sources and specific uptake rates of urea and glutamic acid, an outcome consistent with its relative abundance and the biomass of colonies relative to that of free-living plankton. Further community analysis supports these findings as the CCA was minimally changed when heterotrophic bacteria were added, strongly suggesting that cyanobacteria were the primary drivers of nutrient uptake rates. At the ecosystem level, the onset of dense *Microcystis* blooms, therefore, transforms N cycling whereby most N sources are routed through *Microcystis* colonies (Chen et al., 2012; Peng et al., 2017; Shen et al., 2020; Xie et al., 2024) and associated plankton (Shen et al., 2020; Cai et al., 2024; Xie et al., 2024).

Beyond the *Microcystis* colony fraction, ammonium and nitrate specific uptake were associated with the free-living fraction and other cyanobacteria, including *Aphanizomenon* and *Dolichospermum*, which belong to the order Nostocales (Fogg, 1942; Issa et al., 2014) and can fix N_2_ (Wolk et al., 1994; Hoffman et al., 2014). This highlights a N-uptake-based niche partitioning between *Microcystis* colony-associated communities and other microbes. Diazotrophic cyanobacteria can experience significantly faster growth when utilizing ammonium (Kramer et al., 2022) compared to urea (Jørgensen, 2006). While urea is a key nutrient that can support and promote CHABs dominated by *Microcystis* (Belisle et al., 2016; Chaffin and Bridgeman, 2014; Krausfeldt et al., 2019; this study), other cyanobacteria, including those that fix N_2_, may primarily rely on ammonium to sustain growth, highlighting a niche partitioning with *Microcystis* colony-associated plankton relying on urea and other plankton more reliant on ammonium.

### Conclusion

This study demonstrates that nitrogen assimilation during *Microcystis* blooms differ significantly between colony-associated and free-living plankton communities regarding both community composition and uptake of N. The free-living fraction exhibited lower volumetric uptake rates but higher specific uptake, while conversely the *Microcystis* colony fraction exhibited higher volumetric uptake rates with lower, and therefore less efficient, specific uptake. Both groups primarily used urea as their preferred N source, especially during summer months, while some non-*Microcystis* cyanobacteria relied on ammonium and, to a lesser extent, nitrate. Differences in N assimilation were associated with the trophic status of ecosystems, linked to specific environmental parameters (TN, TP, chl-*a*, microcystin), and microbial interactions. Inverse relationships between volumetric N uptake in the free-living fraction and indicators of trophic state suggested a competition for N with *Microcystis* colonies during CHABs. An inverse relationship between N-specific uptake in the *Microcystis* colony fraction and trophic state indicated a lowered efficiency of uptake by *Microcystis* colonies as blooms intensified, potentially associated with increased microbial competition and/or the limitations of diffusion associated with larger colonies. Further studies are needed to clarify microbial competition for N during CHABs, particularly within and around the matrix of *Microcystis* colonies; however, these results reinforce the need to consider urea alongside inorganic nitrogen forms when evaluating drivers of bloom development and persistence.

## Data Availability

The original contributions presented in the study are publicly available. This data can be found at: https://www.ncbi.nlm.nih.gov/, BioProject ID PRJNA1356834.
