# Supplementary material for: Differential nitrogen assimilation by Microcystis and co-occurring plankton during harmful cyanobacterial blooms across North American Lakes
Source: Front Microbiol. 2026 Apr 24;17:1794100. doi: 10.3389/fmicb.2026.1794100 (PMC13153114; doi:10.3389/fmicb.2026.1794100)

***Supplementary Materials for:***

**Differential nitrogen assimilation by *Microcystis* and co-occurring plankton during harmful cyanobacterial blooms across North American Lakes**

^1^Ann Marie E. Famularo-Pecora, ^1,*^Christopher J. Gobler

^1^School of Marine and Atmospheric Sciences, Stony Brook University, Southampton, NY, 11942, USA

[*christopher.gobler@stonybrook.edu](mailto:*christopher.gobler@stonybrook.edu)


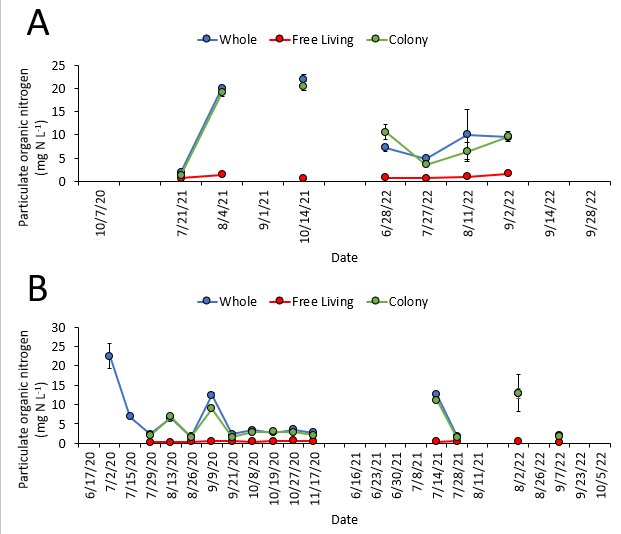


**Supplemental Figure 1.** The average particulate organic nitrogen content of biomass of whole water and fractions from (A) the Lake in Central Park time series study site and (B) the Lake Agawam time series study site from the summers of 2020-2022. Points are means, and error bars are ± 1 S.D.


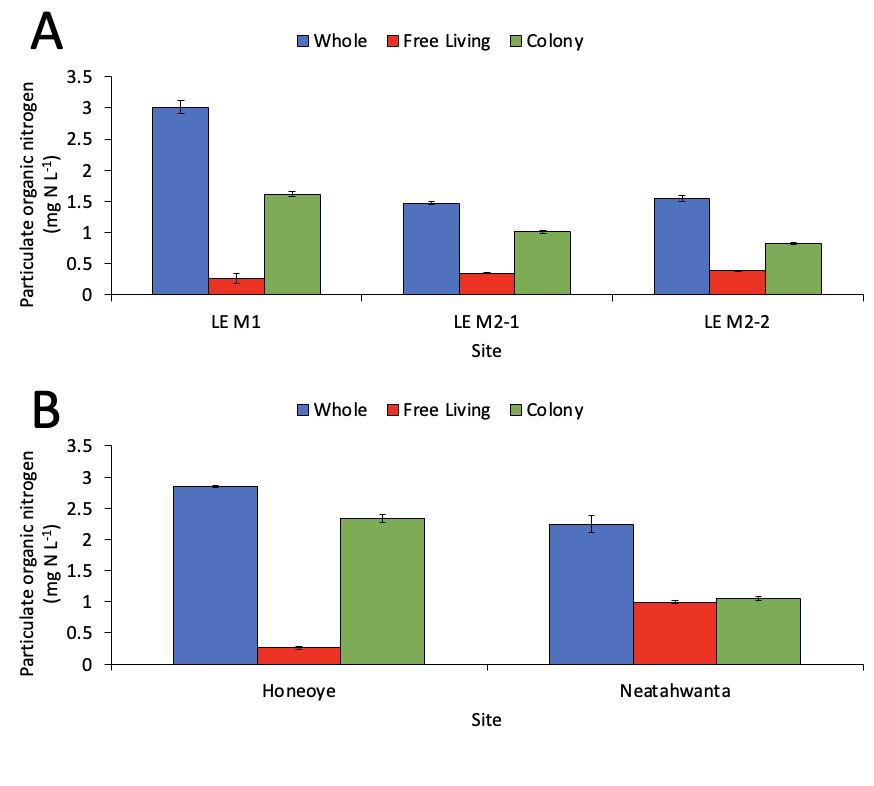


**Supplemental Figure 2.** The average particulate organic nitrogen content of biomass of whole water and fractions from (A) the Lake Erie transect study sites from summers of 2021 (M1) and 2022 (M2), and (B) Honeoye Lake (9/2021) and Lake Neatahwanta (9/2021). Site M2 was sampled on two consecutive dates in 2022, 8/18/2022 (M2-1) and 8/19/2022 (M2-2). Points are means and error bars are ± 1 S.D.


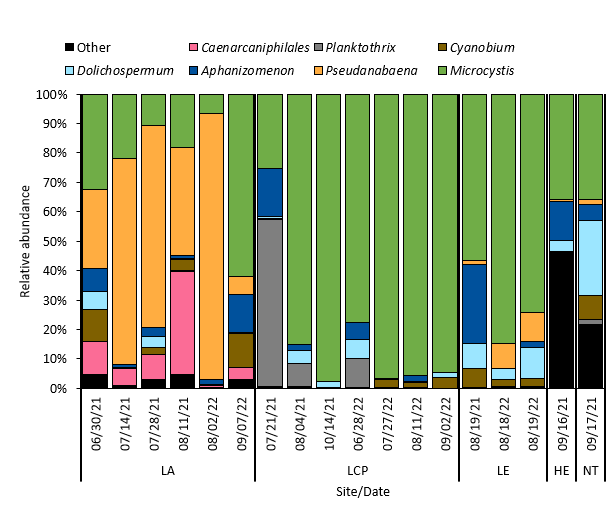


**Supplemental Figure 3.** Time series of the cyanobacterial relative abundance determined via 16S sequencing in the whole water, grouped by lake (Lake Agawam; LA, The Lake in Central Park; LCP, Lake Erie; LE, Honeoye Lake; HE, and Neatahwanta Lake; NT). All low abundant phyla have been grouped into the “other” category.


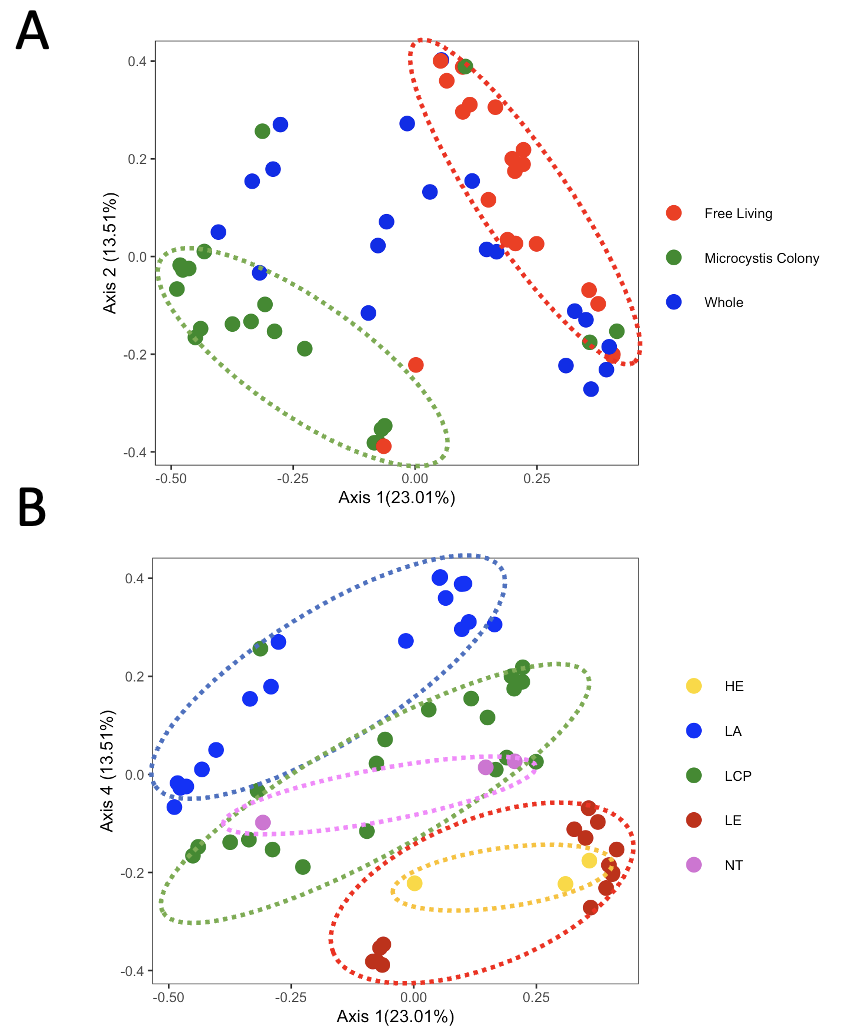


**Supplemental Figure 4.** Principal coordinates analysis (PCoA) conducted on ASV abundances showing the dissimilarity of the heterotrophic bacteria compositions between samples among all lake time series and spatial survey samples from 2021 and 2022. Colors denote the (A) whole water and size fractions, and (B) lake system of the samples (Lake Agawam; LA, The Lake in Central Park; LCP, Lake Erie; LE, Honeoye Lake; HE, and Lake Neatahwanta; NT). Dashed circles indicate significant clustering of the communities.


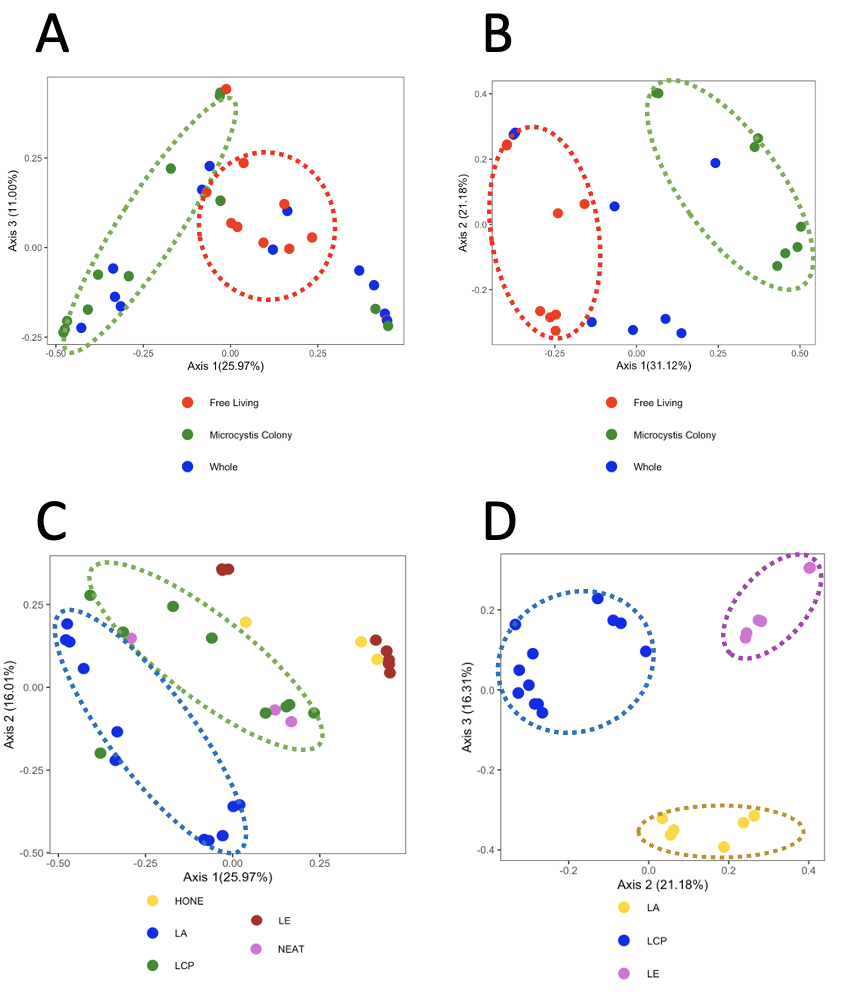


**Supplemental Figure 5.** Principal coordinates analysis (PCoA) conducted on ASV abundances showing the dissimilarity of the heterotrophic bacteria compositions between samples among all lake time series and spatial survey samples from (Left) 2021 and (Right) 2022. Colors denote the (A-B) whole water and size fractions, and (C-D) lake system of the samples (Lake Agawam; LA, The Lake in Central Park; LCP, Lake Erie; LE, Honeoye Lake; HE, and Lake Neatahwanta; NT).. Dashed circles indicate significant clustering of the communities.


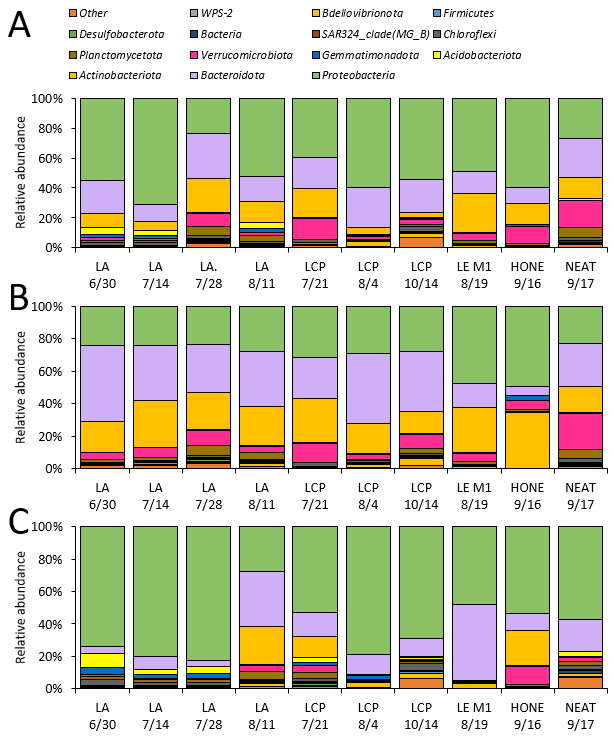


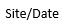


**Supplemental Figure 6.** Time series of 2021 bacteria phylum relative abundance data determined via 16S sequencing in the (A) whole water, (B) free-living fraction, and (C) *Microcystis* colony fraction (Lake Agawam; LA, The Lake in Central Park; LCP, Lake Erie; LE, Honeoye Lake; HE, and Lake Neatahwanta; NT). All low abundance phyla have been grouped into the “other” category.


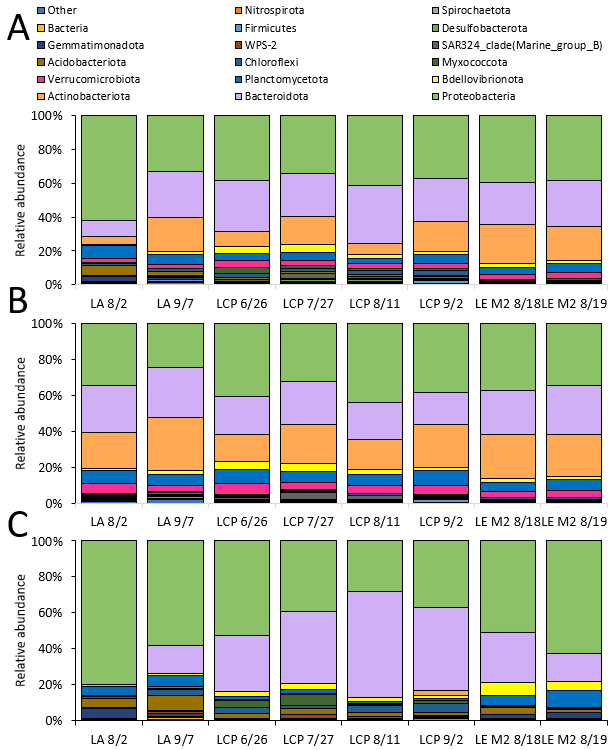


**
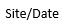
**

**Supplemental Figure 7.** Time series of 2022 bacteria phylum relative abundances determined via 16S sequencing in the (A) whole water, (B) free-living fraction, and (C) *Microcystis* colony fraction (Lake Agawam; LA, The Lake in Central Park; LCP, Lake Erie; LE). All low abundance phyla have been grouped into the “other” category.


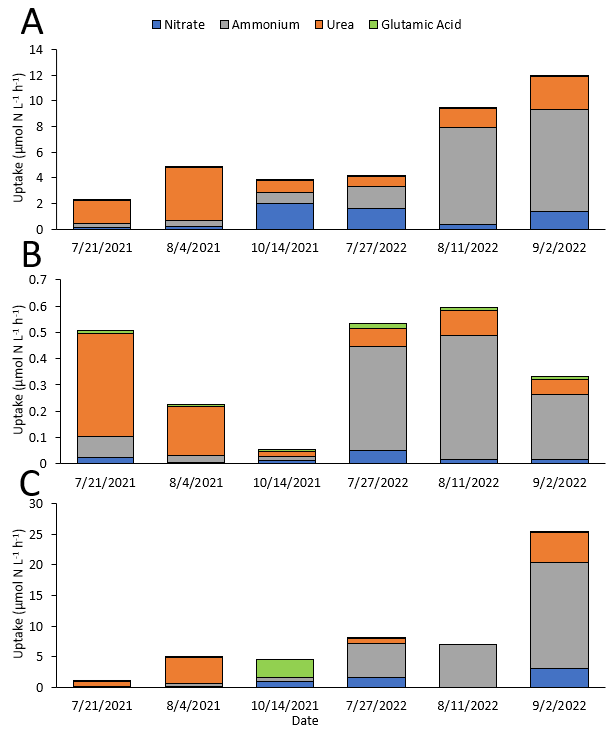


**Supplemental Figure 8.** Nitrogen uptake for the Lake in Central Park by (A) whole water, (B) free-living fraction, and (C) *Microcystis* colony fraction, including nitrate, ammonium, urea, and glutamic acid from dates tested in 2020-2022.


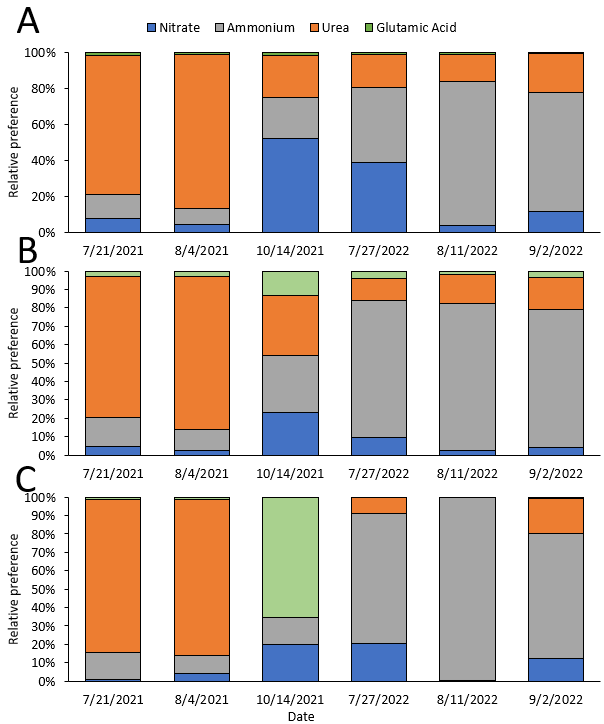


**Supplemental Figure 9.** Relative preference for nitrogen uptake for the Lake in Central Park by (A) whole water, (B) free-living fraction, and (C) *Microcystis* colony fraction, including nitrate, ammonium, urea, and glutamic acid from dates tested in 2020-2022.


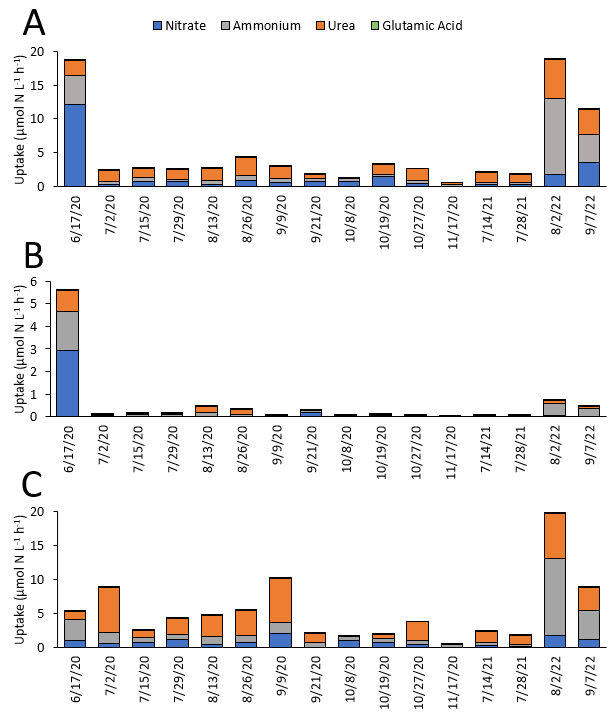


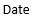


**Supplemental Figure 10.** Nitrogen uptake for Lake Agawam by (A) whole water, (B) free-living fraction, and (C) *Microcystis* colony fraction, including nitrate, ammonium, urea, and glutamic acid from dates tested in 2020-2022.


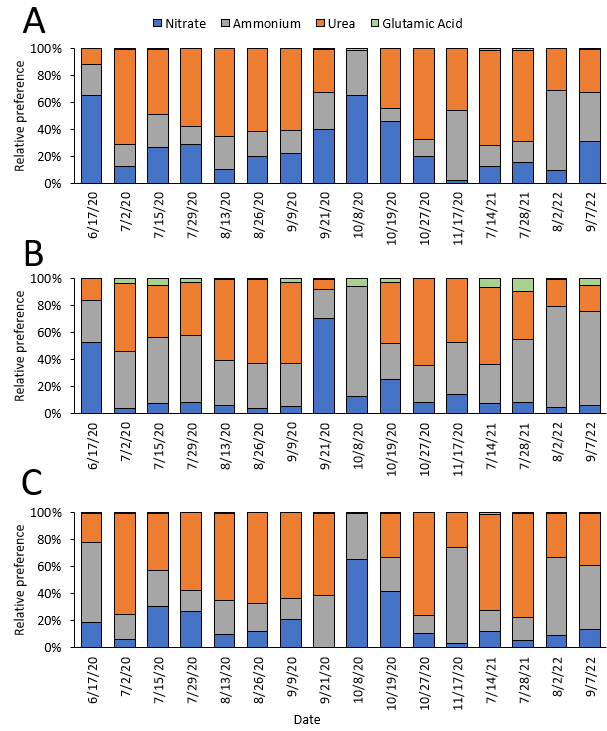


**Supplemental Figure 11.** Relative preference for nitrogen uptake for Lake Agawam by (A) whole water, (B) free-living fraction, and (C) *Microcystis* colony fraction, including nitrate, ammonium, urea, and glutamic acid from dates tested in 2020-2022.


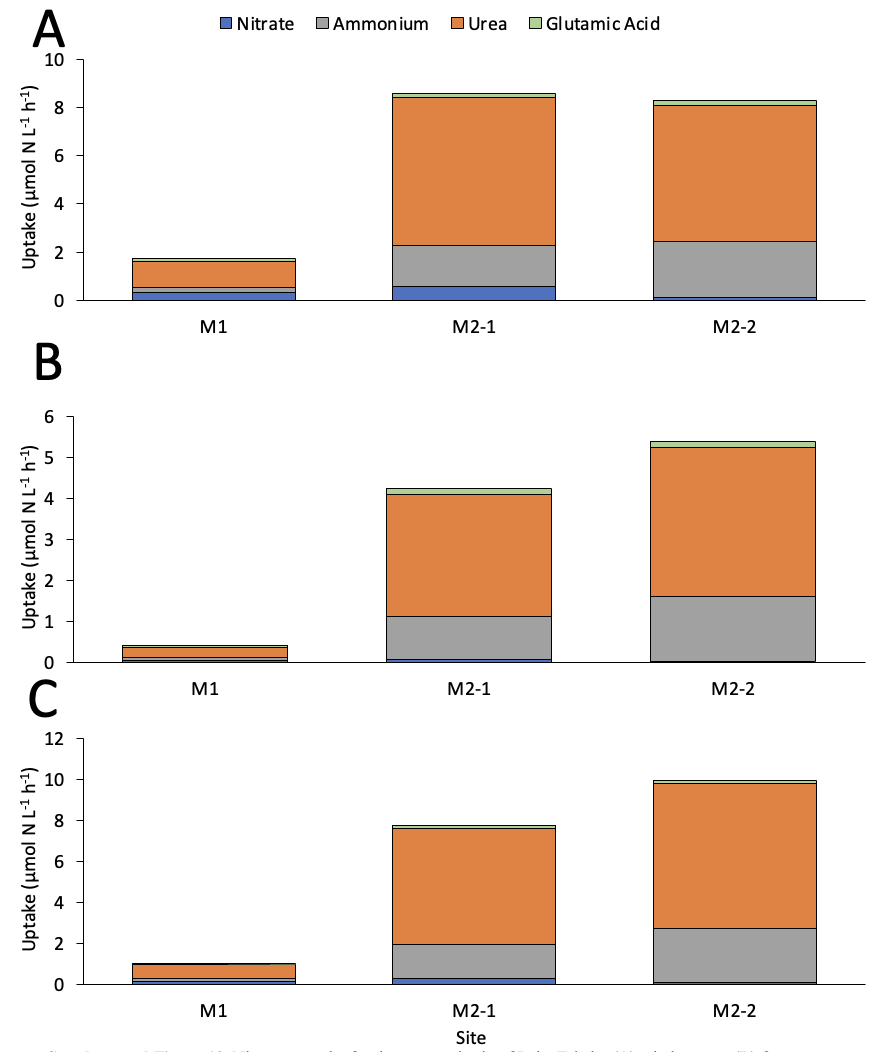


**Supplemental Figure 12.** Nitrogen uptake for the western basin of Lake Erie by (A) whole water, (B) free-living fraction, and (C) *Microcystis* colony fraction, including nitrate, ammonium, urea, and glutamic acid from dates/sites tested in 2021 (M1) and 2022 (M2).


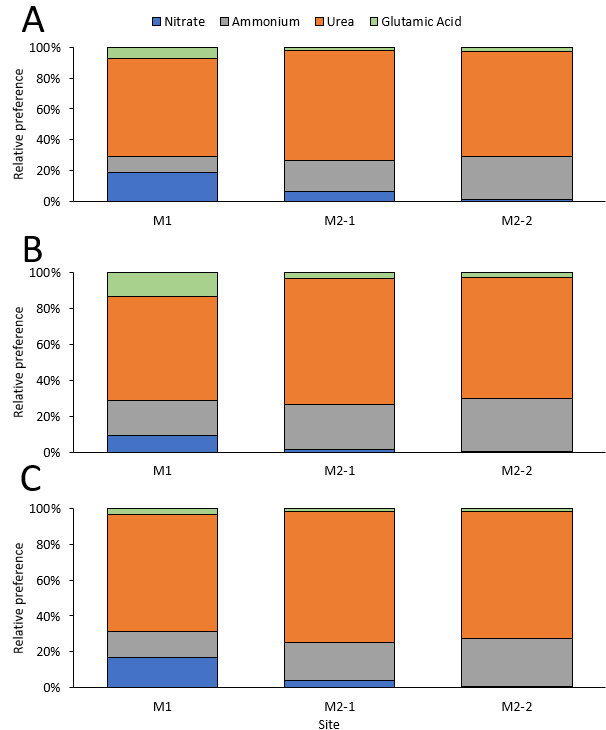


**Supplemental Figure 13.** Relative preference for nitrogen uptake for the western basin of Lake Erie by (A) whole water, (B) free-living fraction, and (C) *Microcystis* colony fraction, including nitrate, ammonium, urea, and glutamic acid from dates/sites tested in 2021 (M1) and 2022 (M2).


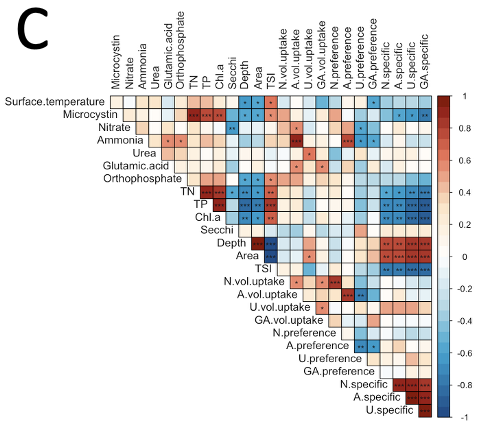

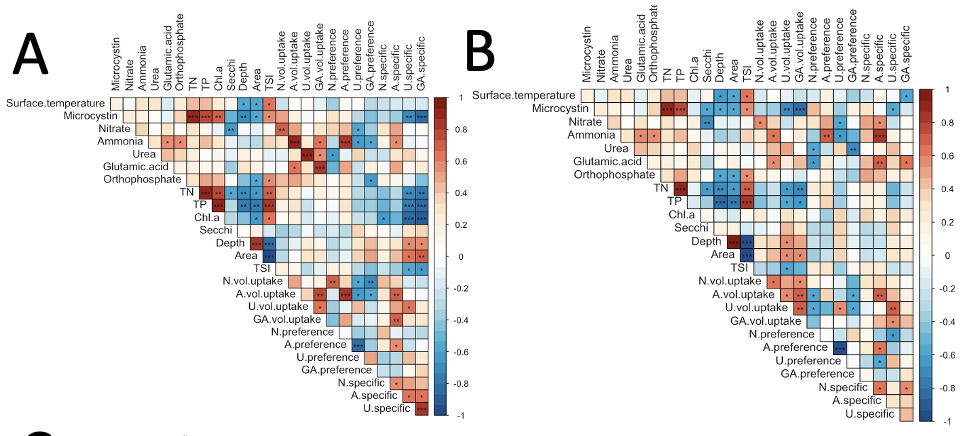


**Supplemental Figure 14.** Total correlation matrices conducted for (A) whole water, (B) free-living fraction, and (C) *Microcystis* colony fraction for 2021 and 2022, including relationships between environmental data, volumetric uptake rates (vol), N-specific uptake rates (specific), and preference. Uptake rates include nitrate (N), ammonium (A), urea (U) and glutamic acid (GA). Gradient indicates direct (red) and indirect (blue) correlations and ranges from -1.0 – 1.0; Asterisks represent significance at *p*<0.05 (*), *p*<0.01 (**), and *p*<0.001 (***).


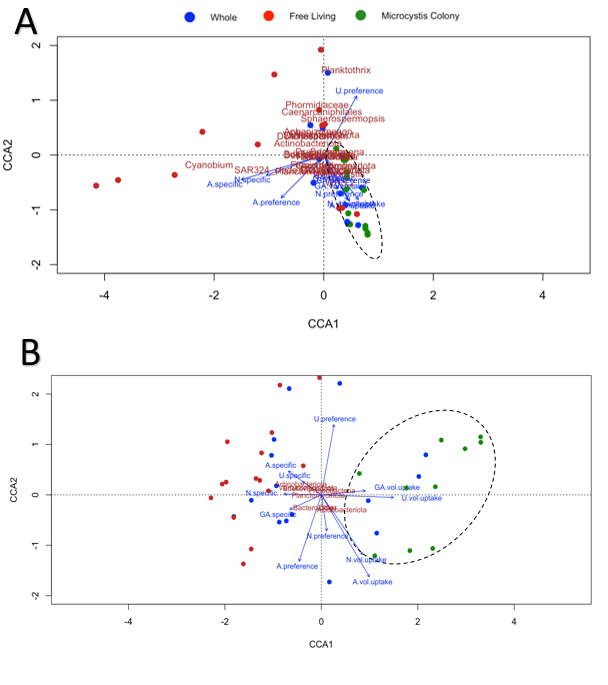


**Supplemental Figure 15.** Canonical correlation analysis (CCA) showing strength (length) and correlation (direction) of uptake rates of interest (bi-plot arrows) to the 16S community clusters for (A) cyanobacterial and heterotrophic bacterial abundances and (B) heterotrophic bacterial abundances, colors denote whole water and fractions. Circles indicate clustering of MCA communities. Proportion explained (A) CCA1: 23.47%, CCA2: 13.88%; (B) CCA1: 50.50%, CCA2: 25.85%.


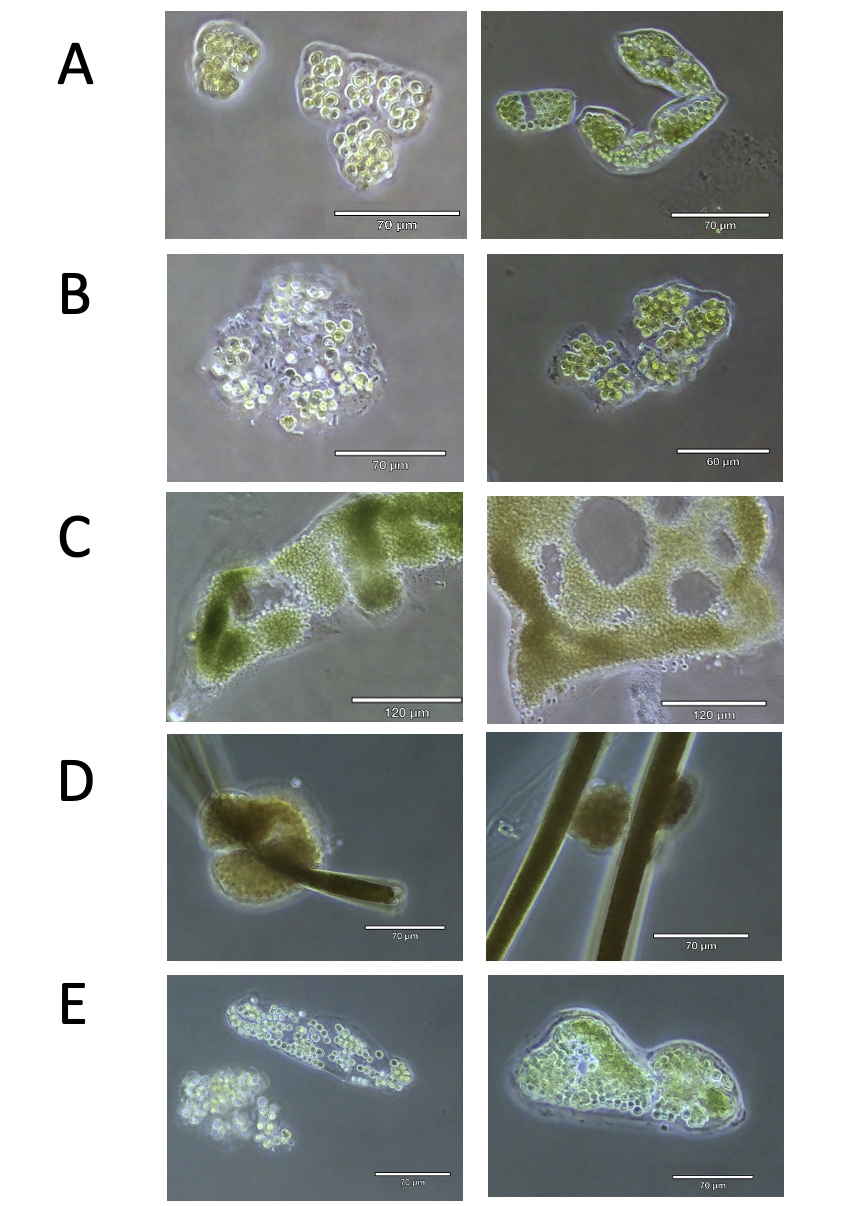


**Supplemental Figure 16.** Microscopic images of *Microcystis* colonies from (A) the Lake in Central Park, (B) Lake Agawam, (C) Lake Erie, (D) Honeoye Lake, and (E) Lake Neatahwanta.

**Supplemental Table 1.** Significance testing for main effects between the whole water communities (WW), free-living fraction (FL), and *Microcystis* colony fraction (MC), and between lakes (Lake Agawam; LA, The Lake in Central Park; LCP, Lake Erie; LE, Honeoye Lake; HE, and Lake Neatahwanta; NT) on the beta diversity of the bacterial communities using PERMANOVA analysis. Communities were analyzed on a per year basis; p value <0.05 used as the threshold for a significant main effect of the fractions on the community structure.


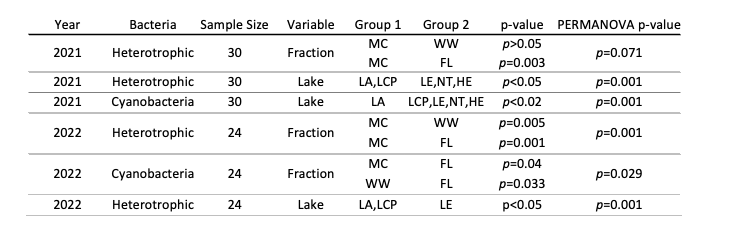

Supplement: Supplementary file 1 [file Supplementary_file_1.docx]
